# Plasma-Derived Atomic Hydrogen Enables Eley–Rideal-Type CO_2_ Methanation at Low Temperatures

**DOI:** 10.1021/jacsau.4c00857

**Published:** 2024-11-19

**Authors:** Dae-Yeong Kim, Yoshinobu Inagaki, Tsukasa Yamakawa, Bang Lu, Yoshiaki Sato, Naoki Shirai, Shinya Furukawa, Hyun-Ha Kim, Satoru Takakusagi, Koichi Sasaki, Tomohiro Nozaki

**Affiliations:** † Department of Mechanical Engineering, Tokyo Institute of Technology, Tokyo 152-8550, Japan; ‡ Division of Applied Quantum Science and Engineering, Hokkaido University, Sapporo 060-8628, Japan; § Institute for catalysis, Hokkaido University N21 W10, Sapporo 001-0021, Japan; # Division of Applied Chemistry, Osaka University, Osaka 565-0871, Japan; ¶ National institute of Advanced Industrial Science and Technology, Tsukuba 305-8569, Japan

**Keywords:** H_2_ activation, nonthermal plasma, CO_2_ methanation, Eley−Rideal-type reactions, Ni/Al_2_O_3_

## Abstract

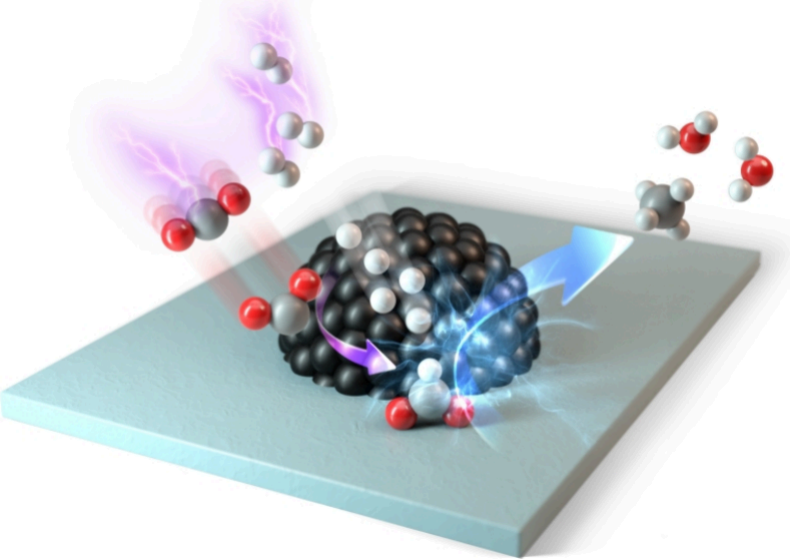

Activating H_2_ molecules into atomic hydrogen and utilizing their intrinsic chemical reactivity are important processes in catalytic hydrogenation. Here, we have developed a plasma-catalyst combined system that directly provides atomic hydrogen from the gas phase to the catalytic reaction to utilize the high energy and translational freedom of atomic hydrogen. In this system, we show that the temperature of CO_2_ methanation over Ni/Al_2_O_3_ can be dramatically lower compared to thermal catalysis. Using a detailed mechanistic study with kinetic studies, laser plasma diagnostics, *in situ* plasma surface characterization, and theoretical calculations, we revealed that plasma-derived atomic hydrogen (PDAH) plays a crucial role in reaction promotion. In particular, PDAH effectively lowers the energy barrier of bidentate formate hydrogenation by translating from the Langmuir–Hinshelwood to the Eley–Rideal-type reaction.

## Introduction

Fossil fuel reserves are decreasing, and excessive use of fossil fuels leads to indiscriminate emissions of CO_2_ into the atmosphere, which has a significant impact on climate change.^1,2^ CO_2_ methanation (CO_2_ + 4H_2_ → CH_4_ + 2H_2_O; Δ*H*_298K_ = −165 kJ/mol) is recognized as an important process for recycling and utilizing CO_2_ by implementing power-to-gas technology.^3,4^ However, although thermodynamically this reaction favors lower temperatures, it must be carried out at high temperatures because, as an eight-electron reaction, kinetically it involves several steps with high energy barriers. These high temperatures increase energy consumption and cause other problems such as the formation of byproducts (CO by reverse water gas shift reaction) and carbon deposition in the catalyst. Therefore, enhancing the low-temperature activity for CO_2_ methanation depends on their ability to recognize and overcome the high energy barrier of the hydrogenation step.

Typically, catalytic hydrogenation occurs by atomic hydrogen generated via the dissociative chemisorption mechanism of H_2_ molecules on the metal surface.^5^ During this process, the adsorbed atomic hydrogen becomes stabilized and immobilized, leading to a decrease in its intrinsic reactivity. Consequently, fully harnessing the chemical reactivity of atomic hydrogen is an inherent obstacle, and strategies for improvement are necessary.

Meanwhile, nonthermal plasma (NTP) is characterized by a nonthermal energy distribution, where the temperature of the electrons (10^4^–10^5^ K) is much higher than the bulk gas temperature (which can be as low as room temperature). Inelastic collisions of these high-energy electrons with ground-state molecules lead to the formation of a large number of activated species.^6−8^ Plasma catalysis, which applies NTP to conventional catalysis, has recently attracted significant attention due to the synergistic effects arising from the interaction between the plasma and the catalyst.^9−16^ Ahmad et al. reported that Ni/Al_2_O_3_ (Ni loading of 10%) exhibited CO_2_ conversion of 60% and CH_4_ selectivity of 97% at 150 °C, which required a much higher temperature of over 300 °C to attain a similar CO_2_ conversion in thermal catalysis.^17^ Chen et al. demonstrated that the 15Ni-20La/Na-BEATA catalyst exhibits a CO_2_ conversion of 85% and CH_4_ selectivity of 97% at temperatures below 150 °C, where it was not active under thermal catalysis.^18^ However, in these studies, the methanation performance was evaluated by the reactor wall temperature and not the catalyst temperature. The catalyst temperature is generally higher than that of the reactor wall, which could lead to an overestimation of catalytic performance under plasma conditions. Additionally, experiments without temperature control are not suitable for kinetic analysis, making it difficult to elucidate the plasma catalytic reaction mechanism. It is necessary to directly measure the catalyst temperature under plasma conditions to accurately evaluate the catalytic performance. Overall, an understanding of the dynamics and reaction mechanisms of gaseous-plasma-activated species on catalyst surfaces is still lacking. Furthermore, the nature of plasma-derived atomic hydrogen (PDAH) remains unclear and has rarely been investigated in detail to assess its reactivity. A combined approach involving kinetic analysis through temperature-controlled experiments,^19^ in situ plasma characterization, and theoretical calculations is desirable for elucidating the reaction mechanisms of plasma catalysts.

We investigated plasma catalytic CO_2_ methanation over Ni/Al_2_O_3_ and discovered the unique reaction behavior of plasma-activated hydrogen corresponding to the Eley–Rideal (E–R, recently also called Langmuir–Rideal^20^)-type reactions in CO_2_ methanation. We performed an in-depth mechanistic study combining kinetic studies, laser plasma diagnostics, in situ plasma characterization, and density functional theory (DFT) calculations to discuss and rationalize how PDAH can efficiently catalyze. In comparison with the thermal conditions, PDAH with high energy and translational freedom activates the E–R-type reaction channel, lowering the energy barrier for the rate-determining step of CH_4_ formation (82.1 → 58.6 kJ/mol). Therefore, while maintaining a high CH_4_ selectivity of >98%, the CO_2_ conversion was improved at lower temperatures below 300 °C.

## Methods

### Catalyst Preparation and Characterization

Ni/Al_2_O_3_ was prepared by the deposition-precipitation method with a loading amound of 6 wt %. Aqueous solution of urea was added dropwise to a vigorously stirred mixture of γ-Al_2_O_3_ (99.9%, Kojundo Chemical Lab) and an aqueous solution of Ni(NO_3_)_2_·6H_2_O (99%, FUJIFILM Wako) in a glass beaker. The mixture was sealed tightly with a plastic film and heated with stirring on a hot stirrer. The temperature of the mixture was kept at 90 °C for 5 h. After deposition, the colorless supernatant was removed and the resulting solid was washed with deionized water three times, followed by drying under reduced pressure. The resulting powder was calcined in air at 500 °C for 1 h and then reduced at 600 °C under a 50 mL/min H_2_ flow for 1 h. The X-ray diffraction (XRD) pattern of Ni/Al_2_O_3_ was recorded by using a MiniFlex 600+D/teX Ultra2 instrument with a Cu Kα X-ray source. High-angle annular dark filed scanning transmission electron microscopy (HAADF-STEM) analysis was conducted using an FEI Talos F200X microscope. As shown in Figure S1, it shows that Ni nanoparticles with an average size of 4.7 nm were evenly dispersed on Al_2_O_3_.

### CO_2_ Methanation Performance

**Figure 1:**
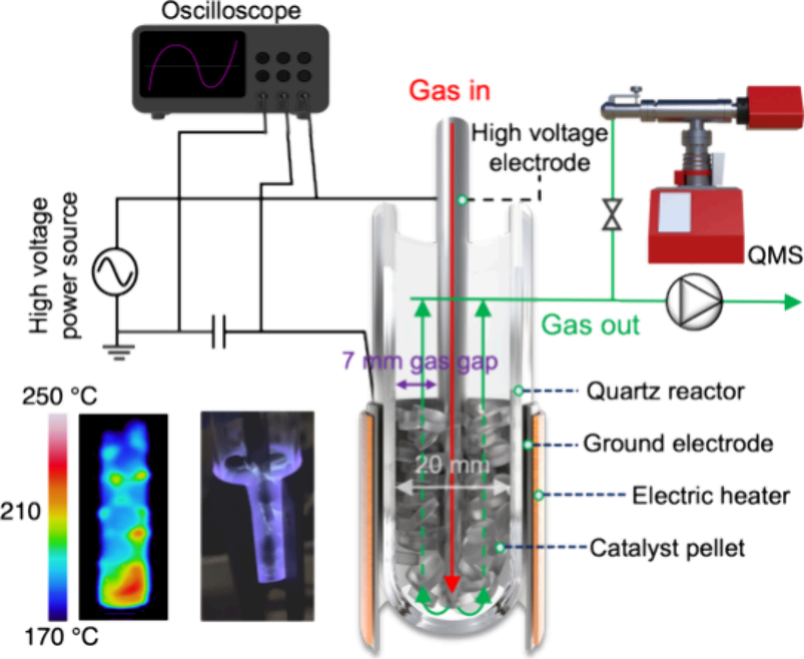
Schematic diagram of the packed-bed DBD reactor. The inset picture shows a DBD generated by a high-voltage power source at 80 kPa.

The CO_2_ methanation performance over Ni/Al_2_O_3_ was evaluated in a packed-bed dielectric barrier discharge (DBD) reactor (Figure 1).

The reactor consists of a quartz tube (20 mm i.d. × 23 mm o.d.), a high-voltage electrode (stainless steel; 1 mm i.d. × 6 mm o.d.), a ground electrode (stainless steel) outside the quartz tube, and a furnace. High-voltage electrodes were placed coaxially along the reactor axis with a 7 mm gas gap for the DBD generation. The powder catalyst was manufactured in pellets (5 mm diameter and 2 mm thickness). Before CO_2_ methanation performance evaluation, 6 g of Ni/Al_2_O_3_ catalyst was reduced at 700 °C for 60 min in 10% H_2_/Ar gas flow (total flow rate = 550 mL/min). Afterward, the catalyst was packaged in a quartz tube for the reaction, and a mixture of H_2_ and CO_2_ was introduced through the tip of the high-voltage electrode. Ar was bypassed in the reactor and was not included in the reaction. The total flow rate and weight hourly space velocity (WHSV) of the mixture of H_2_ and CO_2_ (molar ratio 4:1) were 500 mL/min and 5000 mL/g/h, respectively, under standard temperature and pressure (STP) conditions (25 °C and 101 kPa). The total pressure was fixed at 80 kPa. DBD was generated using AC high-voltage power (Logy Electric; LHV-13AC, Vp-p = +8 to −6 kV, 12 kHz). The applied voltage and average current were measured with an oscilloscope (Agilent Technologies, DSO-X 3014A). The discharge power was obtained by the voltage-charge Lissajous figure method and was fixed at 20 W. The catalyst temperature was measured with an infrared camera coupled with an IR reflector (TH5104, NEC Sanei Instrument, Ltd.). The infrared camera was calibrated with a thermocouple without a DBD. Meanwhile, as CO_2_ methanation (exothermic reaction) progresses, the bottom of the catalyst bed, where the reactant gas first comes into contact with the catalyst, becomes hottest (see the IR camera image in Figure 1). Therefore, we plotted the highest temperature in the catalyst bed to evaluate the CO_2_ methanation performance, not the reactor wall temperature.

Specific energy input (SEI) was calculated by the following formula:
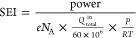


 (mL/min, STP) represents the total gas flow rate at STP conditions (25 °C and 101 kPa). *T* and *P* refer to the catalyst temperature and reaction pressure, respectively. *e* and *N*_A_ represent elementary charge (*C*) and Avogadro number (mol^–1^). SEI represents the mean discharge energy input per molecule (eV/molecule). The calculated SEI for the reaction gas (a mixture of H_2_ and CO_2_) was 0.6 eV/molecule. The gas temperature gradually increases by DBD at 0.6 eV/molecule, and the catalyst temperature is equilibrated with the gas temperature, which has been thoroughly investigated in our previous studies.^21^ Under DBD conditions, when the catalyst temperature reaches a certain point by DBD heating, the CO_2_ methanation is initiated. The onset temperature of CO_2_ methanation is much lower than that of thermal conditions, and CO_2_ methanation exhibits high activity by plasma-excited species even at low temperatures below 300 °C. As a result, the catalyst temperature increases from both the heat generated by CO_2_ methanation and DBD heating, making the use of an external electric heater unnecessary; thus, an external electric heater was not used under DBD conditions. Conversely, under thermal conditions, the CO_2_ methanation activity is low at low temperatures, requiring the use of an external electric heater to raise the catalyst temperature. Quantitative gas analysis was performed by quadrupole mass spectrometry (QMS, Prisma Plus QMG 220). See the Supporting Information for details on obtaining mole flow rates from QMS. The CO_2_ conversion, CH_4_, and CO selectivity were calculated as follows:
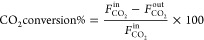

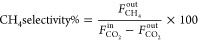

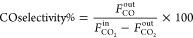

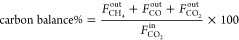


*F* (mol/min) expresses the mole flow rate at STP conditions. Carbon balance typically ranged between 98 and 100% under bother thermal and DBD conditions.

### *In Situ* TIR

The *in situ* TIR of CO_2_ methanation over Ni/Al_2_O_3_ in thermal and plasma catalysis was measured using a DBD flow-type TIR cell (Figure S3). The TIR cell was in the form of a cylindrical glass tube. The high-voltage and ground electrodes (stainless steel; 1 mm diameter) were inserted inside the reactor, and the ground electrode was wrapped in a quartz sheath. The gap between the point-to-point electrodes was 10 mm. A high-voltage power supply was connected to the reactor, and the discharge power and frequency for all catalyst tests were 0.002 W and 19 kHz, respectively, and kept constant. The catalyst was powdered (50 mg), put evenly in a disc kit, compressed with a pressure press, and manufactured in the form of a pellet (10 mm diameter, 1 mm thickness). After that, the catalyst pellet was fixed in a glass holder and inserted 5 mm downstream from the plasma discharge zone. The *in situ* TIR spectra was collected using a Fourier transform infrared spectrometer (FTIR, Jasco FTIR-6100) equipped with a mercury cadmium telluride (MCT) detector with a spectral resolution of 4 cm^–1^. Spectra were recorded every 60 s. The catalyst pellet was reduced at 700 °C under a 10% H_2_/Ar flow for 1 h before measurement. Then, CO_2_ methanation was performed by flowing CO_2_ (10 mL/min) + H_2_ (40 mL/min) diluted in Ar (100 mL/min). Ar was used as a balance gas to dilute the reaction gas and avoid signal saturation of the IR spectra. For each measurement, the total pressure was fixed at 80 kPa.

### *In Situ* XAFS

The S Ni K-edge XAFS measurements of Ni/Al_2_O_3_ were carried out at the BL9A beamline of the Photon Factory (PF) in the Institute of Material Structure Science (IMSS) of High Energy Accelerator Research Organization (KEK). The storage ring energy and ring current were 2.5 GeV and 450 mA, respectively. X-rays were monochromatized with a Si(111) double-crystal monochromator that was focused using a pair of bent conical mirrors. The X-ray beam size was 0.5 mm (horizontal) × 0.3 mm (vertical). The spectra were recorded in transmission mode using the same cell as that for *in situ* TIR (Figure S3). The XANES spectra were normalized to their edge height after background subtraction. The EXAFS spectra were analyzed using the RIGAKU REX2000 software.^22^ The EXAFS oscillations (χ(*k*)) were extracted using a spline smoothing method and normalized to their edge height after background subtraction, where *k* is the photoelectron wavenumber and calculated from the photon energy, *E*, using eq 1.
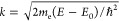
1

*E*_0_ and *m*_e_ are the threshold energy and electron mass, respectively. The quantity of *k*^3^χ(*k*) in the *k*-range of 3.0–13.0 Å^–1^ was Fourier transformed into R-space, and the peak in the transform was filtered (the filtered R-range was 1.32–2.64 Å). Then, an inverse Fourier transform was applied to convert the filtered peak back to *k*-space. The Fourier-filtered data were then analyzed with a curve-fitting technique using the following theoretical EXAFS values: eqs 2 and 3.
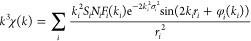
2
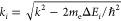
3Here, *S_i_*, *N_i_*, σ_*i*_, *r_i_*, and Δ*E*_*i*_ are the amplitude reduction factor, coordination number, Debye–Waller factor, bond distance of the ith bond, and energy shift in the origin of the photoelectron kinetic energy of the ith bond, respectively. In this study, one shell fitting using the Ni–Ni shell was carried out (*i* = 1 for the Ni–Ni shell). The backscattering amplitude, *F*_1_ (*k*_1_), and phase shift, φ_1_(*k*_1_) were obtained from Ni foil at 300 K for the first shell analysis.^23^ The amplitude reduction factor, *S*_1_, was estimated to be 1.00 ± 0.03. The fitted parameters were *N*_1_, σ_1_, *r*_1_, and Δ*E*_1_. The error of these four parameters was estimated using the Hamilton ratio test with a significance level of 0.317.^24^ A goodness of the fit between the observed and calculated *k*^3^χ(*k*) was evaluated using the *R*-factor defined as eq 4.
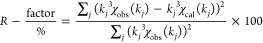
4

### Computational Details

Periodic DFT calculations were performed using the CASTEP code^25^ with Vanderbilt-type ultrasoft on the fly generated (OTFG) pseudopotentials as well as the revised version of the Perdew–Burke–Ernzerhof exchange–correlation functional based on the generalized gradient approximation.^23^ The plane-wave basis set was truncated at a kinetic energy of 500 eV. A 0.1 eV Fermi smearing (0.1 eV) was used. The Tkatchenko–Scheffler method was employed to analyze dispersion correlations with a scaling coefficient of *s*_R_ = 0.94 and a damping parameter of *d* = 20.^26^ Spin polarization was considered for all of the calculation. The reciprocal space was sampled using a *k*-point mesh with a spacing of 0.04 Å^–1^, as generated by the Monkhorst–Pack scheme.^27^ The unit cell size of the face-centered cubic Ni was first optimized (lattice constant: *a* = *b* = *c* = 3.45986 Å), followed by modeling the slab structure and surface relaxation with the size of the supercell fixed. The slab model was constructed using a Ni(111)–(3 × 4) structure with a thickness of six atomic layers with 13 Å of vacuum spacing. Geometry optimizations and TS searches were performed on supercell structures using periodic boundary conditions without fixing any atoms (optimized cell size: *a* = 8.47489 Å, *b* = 7.33947 Å, *c* = 22.98775 Å, α = β = γ = 90°; the fractional coordinates of each Ni atom are listed in Table S3). The convergence criteria for structural optimization and energy calculation were set at (a) a self-consistent field tolerance of 1.0 × 10^–6^ eV per atom, (b) an energy tolerance of 1.0 × 10^–5^ eV per atom, (c) a maximum force tolerance of 0.05 eV Å^–1^, and (d) a maximum displacement tolerance of 1.0 × 10^–3^ Å. The transition state (TS) search was performed using the complete linear synchronous transit/quadratic synchronous transit method.^28,29^ The convergence criterion for the TS calculations was set at root-mean-square forces on an atom tolerance of 0.1 eV Å^–1^.

## Results and Discussion

### CO_2_ Methanation Performance and Kinetic Study

**Figure 2:**
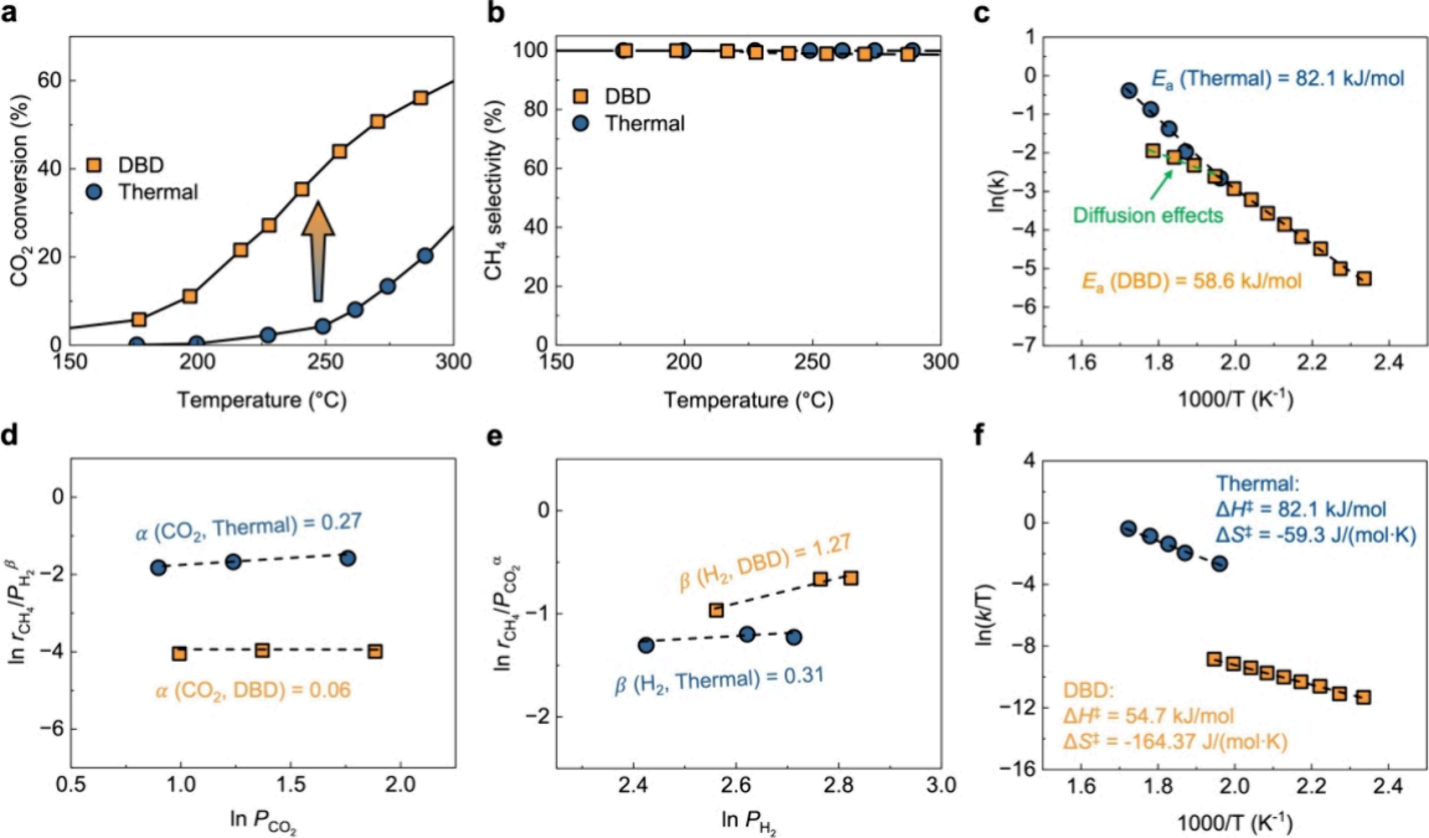
Catalytic performance and kinetic study for CO_2_ methanation under thermal and DBD conditions over Ni/Al_2_O_3_. (a) Temperature-dependent CO_2_ conversion, (b) CH_4_ selectivity, (c) Arrhenius plots, and reaction order of (d) CO_2_ and (e) H_2_. (f) Eyring plots. Reaction orders with respect to reactant under thermal and DBD conditions were determined at 260 and 200 °C, respectively. Total flow rate = 500 mL/min (STP); H_2_/CO_2_ = 4; WHSV = 5000 cm^3^/g/h (STP); pressure = 80 kPa; and SEI = 0.6 eV/molecules. Kinetic analyses were performed in the packed-bed DBD reactor (Figure 1) without the use of Ar.

Figure 2a shows the temperature-dependent CO_2_ conversion for Ni/Al_2_O_3_. Compared to the thermal conditions, the onset temperature of CO_2_ methanation under the DBD conditions is reduced by more than 50 °C, and the CO_2_ conversion is promoted at low temperatures below 300 °C, while maintaining a high CH_4_ selectivity of >98% (Figure 2b). In particular, at about 230 °C, the CO_2_ conversion is 27.2% for DBD conditions, which is an improvement of more than 11-fold compared with thermal conditions (2.3%). Meanwhile, even if the CO_2_ conversion is promoted under DBD conditions compared to thermal conditions, it is much lower than that under equilibrium (Figure S4), so the influence on the reverse reaction can be ruled out. Moreover, because the methanation reaction is operated at a much lower temperature than that of steam CH_4_ reforming (i.e., the reverse reaction of methanation), the effect of DBD on already generated products is ignored.^30^ To better describe the promoted catalytic activity by plasma, we estimated the activation energy (*E*_a_) of the reaction was estimated. The detailed kinetic analysis is described in the Supporting Information. To obtain the differential mass normalized reaction rate under both thermal and DBD conditions, the Arrhenius plot was constructed based on data obtained from 220 to 290 °C for thermal conditions and 150 to 220 °C for DBD conditions, respectively (Figure 2c). The *E*_a_ under thermal conditions is 82.1 kJ/mol, and this value is in line with other previous reports for Ni/Al_2_O_3_.^31−33^ The estimated *E*_a_ under DBD conditions is 58.6 kJ/mol, which is equivalent to the reported thermal catalytic CO_2_ methanation over the Ru catalyst,^34^ suggesting that the energy barrier for CH_4_ formation can be efficiently lowered by plasma. Meanwhile, at high temperatures (above 250 °C), the slope is smaller than that at low temperatures, indicating a diffusion-controlled reaction. We also determined the reaction orders of CO_2_ (Figure 2d) and H_2_ (Figure 2e). First, in the case of thermal conditions, CO_2_ and H_2_ show nearly zero-order dependence. It is well known from kinetic and modeling studies that CO_2_ methanation on Ni/Al_2_O_3_ under thermal conditions proceeds via the Langmuir–Hinshelwood (L-H)-type reactions.^31,33,35^ Furthermore, the nearly zero-order reaction of CO_2_ and H_2_ is in good agreement with previous reports, providing key evidence that CO_2_ methanation under thermal conditions proceeds via the L–H-type reactions. For DBD conditions, the reaction order of CO_2_ decreases clearly compared to the thermal conditions, which means faster adsorption on the surface by plasma-activated CO_2_.^13,36,37^ Interestingly, a first-order dependence on the reaction order of H_2_ is observed under DBD conditions. The difference in the reaction order of H_2_ reflects that plasma-activated H_2_ is an important factor in the promotion of plasma-catalytic CO_2_ methanation over Ni/Al_2_O_3_. Meanwhile, atomic hydrogen can be easily generated by hydrogen dissociation through electron collisions and is the most abundant radical among plasma-activated hydrogen.^38,39^ The E–R-type and hot-atom-type channels can be created by PDAH.

The trade-off between these two reactions strongly depends on the coverage of the surface intermediates.^40^ The zero-order reaction of CO_2_ observed under both thermal and plasma conditions indicates high adsorbed intermediate coverage derived from the formation of CO_2_. Therefore, the plausible interpretation under DBD conditions is that the catalyst surface is covered with an adsorbed intermediate derived from CO_2_ (such as carbonates), and CH_4_ is formed through a direct reaction of these with PDAH, i.e., the E–R-type reactions. In that case, the reaction rate for CH_4_ generation is proportional to H_2_ partial pressure, or reaction order for H_2_ could be close to one.^41^ Meanwhile, the entropy change of the E–R-type reactions takes a large negative value because the high translational freedom of the gas molecules is lost via the surface reaction. Therefore, we further estimated the activation parameters via an Eyring plot (Figure 2f). Activation enthalpy (Δ*H*^‡^) and entropy (Δ*S*^‡^) values were both decreased in the DBD conditions compared to those under the thermal conditions. In particular, the sharp decrease in the Δ*S*^‡^ value provides clear support that the rate-determining step has been changed from the L–H to the E–R-type reactions.

### Lifetime of PDAH

The above results suggest that PDAH creates a new reaction channel with a much lower activation barrier, which, in turn, significantly enhances CO_2_ methanation. In other words, this indicates facile access of PDAH to the catalyst surface. Therefore, to investigate the lifetime of PDAH in the gas phase, we examined the spatial afterglow of an atmospheric pressure DBD with the mixture of H_2_, CO_2_, and Ar (see the Supporting Information for details).

Figure S7 shows an image that represents the spatial distribution of the PDAH density measured by two-photon absorption laser-induced fluorescence (TALIF) using a 205.08 nm dye laser. Figure S7 shows time-averaged H densities. The absolute density of PDAH was on the order of 10^15^ cm^–3^ at 101.3 kPa and 450 K. The axial decay of the PDAH density was gentle, and the characteristic decay length was approximately 35 mm. Assuming that PDAH decay is due to the three-body recombination (H + H + M = H_2_ + M), the lifetime of PDAH is estimated as on the order of 4 ms, corresponding to 35 mm decay length under the given gas jet velocity. It is noteworthy that the lifetime of PDAH in the Ar/H_2_/CO_2_ mixture is the same order as that of Ar/H_2_; PDAH scavenging by CO_2_, such as CO_2_ + H = CO + OH, is ignored in Ar/H_2_/CO_2_ plasma (Figure S7a), ensuring a sufficient amount of H supply to the catalysts.

### *In Situ* TIR

**Figure 3:**
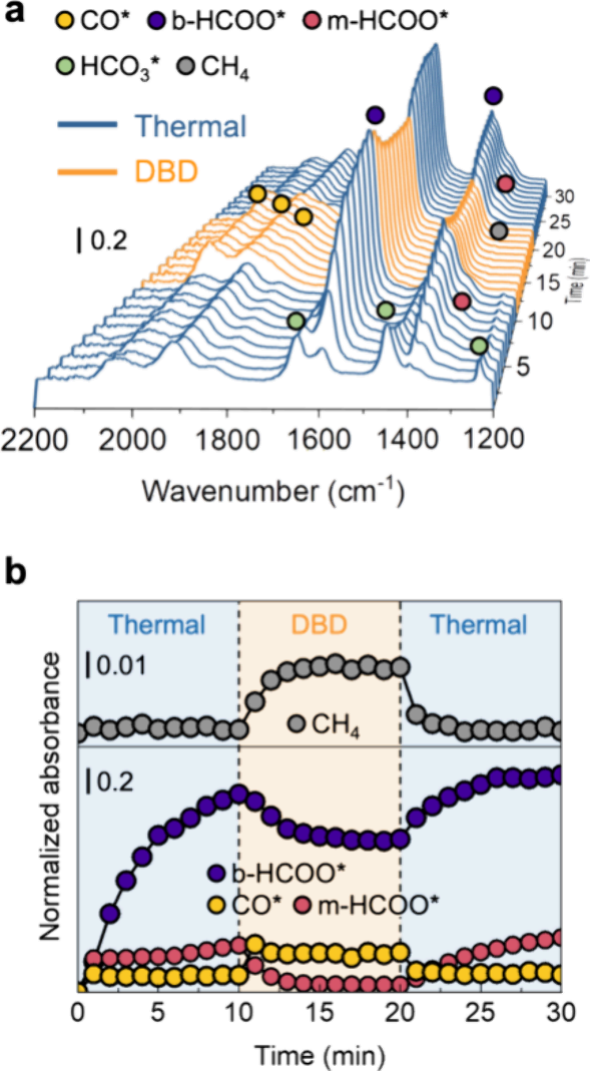
Probing the surface species during the methanation of CO_2_ over Ni/Al_2_O_3_. (a) *In situ* TIR spectra under both thermal and DBD conditions at 210 °C. (b) Normalized absorbance of generated b-HCOO* (1595 cm^–1^), m-HCOO* (1662 cm^–1^), and CO* and CH_4_ (3015 cm^–1^) corresponding to (a). A CO_2_ + H_2_ mixture (H_2_/CO_2_ = 4) was introduced at a constant flow rate. The reaction conditions are switched from thermal to DBD to thermal every 10 min.

We explored the surface adsorbed species via *in situ* TIR, where the DBD is generated in the TIR cell. It was conducted at 210 °C, where there was a significant difference in the CO_2_ conversion between thermal and DBD conditions (Figure 2a). The assignments of the peaks in the TIR spectra of surface species are summarized in Table S1. The spectra were recorded every 1 min. Figure 3a shows the time-dependent change of the TIR spectra obtained during CO_2_ methanation (H_2_/CO_2_ = 4). For the thermal conditions during the first 10 min, appreciable peaks assigned to the CO species adsorbed on metallic Ni (denoted as CO*) at 2060 to 1830 cm^–1^ and the bicarbonate species (HCO_3_*) at 1649, 1446, and 1230 cm^–1^ are observed as the reaction begins. The absorbance of HCO_3_* gradually decreases with time, while the peaks attributed to the bidentate formate species (b-HCOO*) at 1595, 1392, and 1378 cm^–1^ and the monodentate formate species (m-HCOO*) at 1662 and 1324 cm^–1^ gradually increase. This suggests that b-HCOO* and m-HCOO* are formed from the HCO_3_*hydrogenation.^42^ Under the thermal conditions, we did not detect CH_4_ peaks at 1304 cm^–1^ (Figure 3a) and 3015 cm^–1^ (Figure S8), suggesting that the observed surface species have a low activity for further hydrogenation to CH_4_. Upon switching to the DBD conditions, a decrease in b-HCOO* and m-HCOO* is observed along with an increase in CO*. At the same time, an increase in CH_4_ peaks is observed at 1304 cm^–1^ (Figure 3a) and 3015 cm^–1^ (Figure S8). Meanwhile, no obvious gaseous CO (2250–2000 cm^–1^) was detected, which was attributed to limited plasma CO_2_ dissociation.^36^ Continuing, when switching from the DBD to the thermal conditions again, b-HCOO* (Figure 3a) and m-HCOO* (Figure S9) increase, and CO* decreases. At this moment, the CH_4_ peaks disappear. These results suggest that CO*, m-HCOO*, and b-HCOO* may be involved in CH_4_ formation.

**Figure 4:**
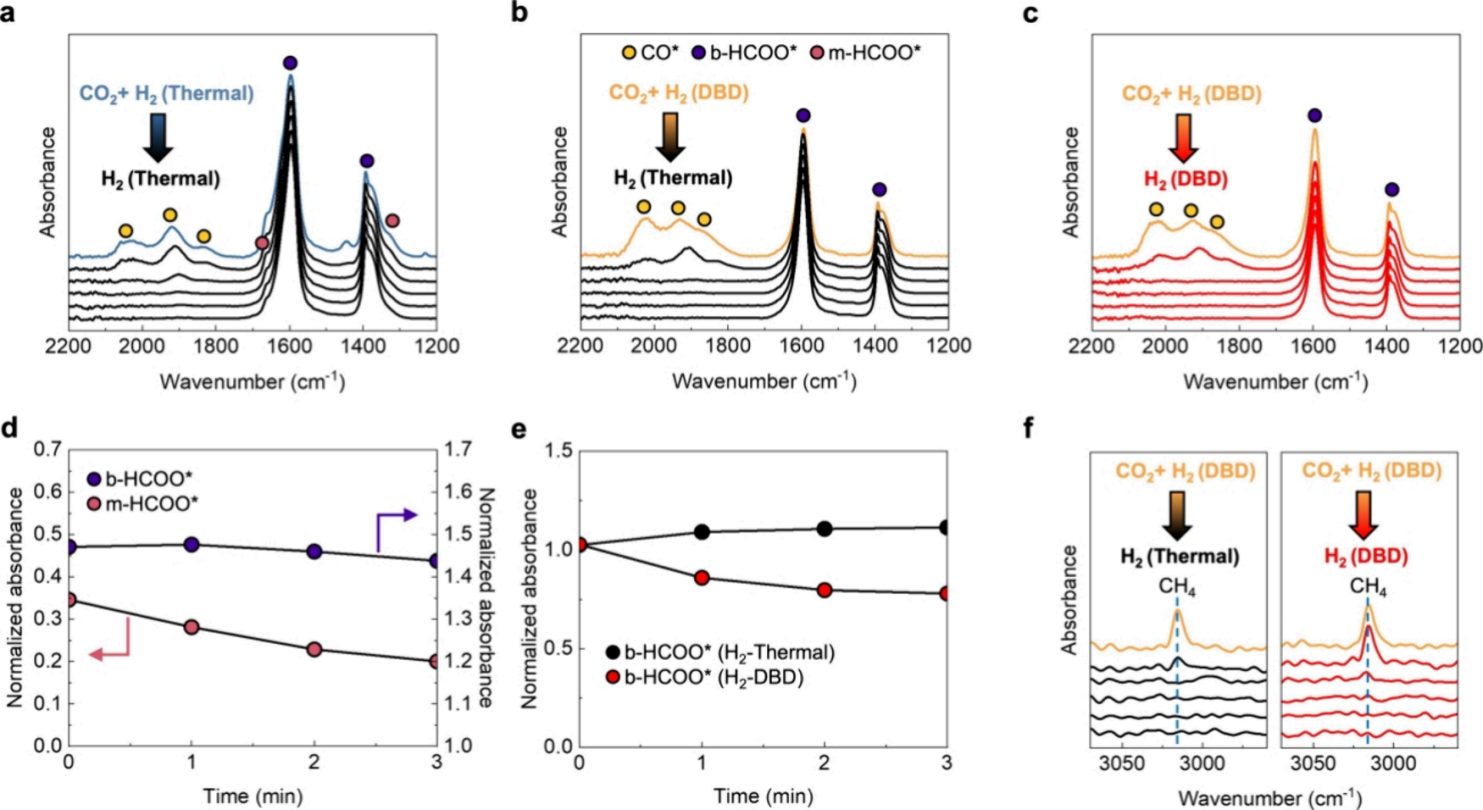
Determination of the reaction pathway of plasma catalytic CO_2_ methanation promotion over Ni/Al_2_O_3_. *In situ* TIR spectra at 210 °C after switching feed gas from (a) A CO_2_ + H_2_ mixture (H_2_/CO_2_ = 4) under thermal conditions to H_2_ under thermal conditions, (b) CO_2_ + H_2_ mixture (H_2_/CO_2_ = 4) under thermal conditions to H_2_ under DBD conditions and (c) CO_2_ + H_2_ mixture (H_2_/CO_2_ = 4) under DBD conditions to H_2_ under DBD conditions. Normalized absorbance of (d) b-HCOO* (1595 cm^–1^) and m-HCOO* (1662 cm^–1^) corresponding to (a) and (e) b-HCOO* (1595 cm^–1^) corresponding to (b) and (c). (f) CH_4_ peak corresponding to (b) and (c).

Subsequently, we performed a series of *in situ* transient TIR spectroscopy to closely examine the respective contributions of CO*, m-HCOO*, and b-HCOO* in the CH_4_ formation. As shown in Figure 4a, when switching the feed gas from the CO_2_ + H_2_ mixture under thermal conditions to H_2_ under thermal conditions, CO* and m-HCOO* decrease over time. In contrast, the b-HCOO* concentration remained almost unchanged (Figure 4d). It suggests that the origin of CO* is due to m-HCOO hydrogenation. Compared with thermal conditions during H_2_/CO_2_ flow in Figure 4a, the absorbance of CO* is stronger during CO_2_ + H_2_ mixture under the DBD conditions in Figure 3b, where m-HCOO* is not detected. This indicates that the m-HCOO* hydrogenation to CO* is promoted by PDAH. In Figure 4a, the presence of CO* is still detected 2 min after the feed gas is switched from H_2_/CO_2_ to H_2_. On the other hand, in Figure 3b, despite the stronger absorbance of CO*, CO* is not detected 2 min after switching to H_2_. Noting that m-HCOO* is not detected during the flow of the CO_2_ + H_2_ mixture under the DBD conditions (Figure 4b), the relatively slow decrease of CO* after switching to H_2_ in Figure 3a may be due to the m-HCOO* hydrogenation to CO*. This provides further evidence that the origin of CO* is m-HCOO* hydrogenation and that CO* is then hydrogenated to CH_4_. Figure 4c shows the TIR spectra when the feed gas is switched from a CO_2_ + H_2_ mixture under DBD conditions to H_2_ under DBD conditions. Upon switching to H_2_ under thermal conditions (Figure 4b) and DBD conditions (Figure 4c), the level of CO* decreases rapidly. Meanwhile, b-HCOO* hardly changed during the H_2_ flow under thermal conditions, while b-HCOO* decreased for 2 min during the H_2_ flow under DBD conditions (Figure 4e). As shown in Figure 4f, the corresponding CH_4_ peak, when switching from a CO_2_ + H_2_ mixture flow under DBD conditions to H_2_ flow under thermal conditions, shows a rapid decrease, whereas when switching to H_2_ under DBD conditions, the CH_4_ peak is observed even after 2 min. This indicates that b-HCOO* is also involved in CH_4_ formation and that the b-HCOO* hydrogenation is also promoted by PDAH. In this study, both CO and b-HCOO pathways coexist in the methanation reaction, resulting in a high CH_4_ selectivity of >98%, as shown in Figure 2b. Furthermore, we investigated the kinetics of b-HCOO* hydrogenation through *in situ* TIR spectra, which are shown in Figure S10. The activation energy (*E*_a_*) of the b-HCOO* hydrogenation reaction obtained under both thermal and DBD conditions are 84.0 and 60.8 kJ/mol, respectively (Figure S10c), which is in good agreement with the values of *E*_a_ in Figure 2c. In other words, the important rate-determining intermediate for CH_4_ formation is b-HCOO*, which explains well that the energy barrier of the rate-determining step can be effectively lowered by PDAH.

### *In Situ* XAFS

The Ni K-edge XANES and EXAFS spectra measured during CO_2_ methanation under thermal and DBD conditions showed no significant difference (Figure S11). The curve fitting results of the EXAFS spectra shown in Table S2 and Figure S12 indicated that all the structural parameters such as coordination number (*N*), bond distance (*R*), and Debye–Waller factor (σ) were similar between thermal and DBD conditions within the margin of error, suggesting that no catalyst restructuring was induced by DBD. It should be noted that there was no catalyst heating by DBD as the σ^2^ value did not change within the margin of error.^43^ Thus, Ni/Al_2_O_3_ did not undergo any structural changes or heating that would affect the plasma catalytic CO_2_ methanation.

### DFT Calculations

Experimental results showed that PDAH could significantly promote CO_2_ methanation by directly reacting with b-HCOO*, that is, via E–R-type reactions. Therefore, we performed DFT calculations to clarify the reaction channel of PDAH with b-HCOO*. Using Ni(111) as a model catalyst, a molecular description of the formation pathway of CH_4_(*g*) was obtained by performing a direct attack of atomic hydrogen to b-HCOO* at 200 °C. The fractional coordinates of Ni atoms in the optimized bare supercell are presented in Table S3. The calculated activation and reaction parameters are presented in Tables 1 (Δ*H*^‡^, Δ*H*, Δ*S*^‡^, and Δ*S*) and S4 (Δ*E*^‡^, and Δ*E*). Also, the specific configurations of the initial (IS), transition (TS), and final state (FS) in each elementary step are summarized in Figure S13.

**Table 1:** Reaction Scheme of CO_2_ Methanation over Ni(111) in Eley–Rideal-Type Reactions and the Corresponding Enthalpies and Entropies at 200 °C^a^

		Δ*H*^‡^	Δ*H*	Δ*S*^‡^	Δ*S*
step	chemical equation	(kJ/mol)		(J/mol·K)	
(1)	b-HCOO* + H(g) → e-HCOOH*	29.9	–111.1	–162.4	–107.4
(2)	e-HCOOH* → s-HCOOH*	24.2	24.1	–2.7	–2.7
(3)	s-HCOOH* + H(g) → h-CHO* + H_2_O(g)	41.6	–311.8	–156.2	39.7
(4)	h-CHO* + H(g) → br-CH_2_O*	15.2	–207.5	–2.4	–111.9
(5)	br-CH_2_O* + H(g) → br-CH_2_OH*	11.3	–228.8	–19.4	–87.2
(6)	br-CH_2_OH* + H(g) → h-CH_2_* + H_2_O(g)	48.4	–275.5	–153.9	56.9
(7)	h-CH_2_* + H(g) → h-CH_3_*	6.0	–238.7	2.7	–96.3
(8)	h-CH_3_* + H(g) → CH_4_(*g*)	∼0	–261.3		3.1

a Abbreviation of adsorption configuration; g: gas-phase, b: bidentate, e: end-on, s: side-on, h: hollow, br: bridge.

Starting from the b-HCOO* protonation to e-HCOOH* (1) and the following tilting to s-HCOOH* (2), CH_4_(*g*) is finally formed through stepwise E–R-type reactions with an atomic hydrogen via s-HCOOH* dehydroxylation (3), h-CHO* hydrogenation (4), h-CH_2_O* protonation (5), br-CH_2_OH* dihydroxylation (6), h-CH_2_* hydrogenation (7), and h-CH_3_* hydrogenation (8). These steps are classified into two types: simple addition of H to C (hydrogenation) or O (protonation) moieties and protonation-initiated dihydroxylation. Interestingly, the activation enthalpy of each step was surprisingly low (Δ*H*^‡^: 0–48.4 kJ/mol) unlike typical chemical reactions. Considering the Bro̷nsted–Evans–Polanyi rule, this should be attributed to the greatly negative reaction enthalpy (Δ*H*: typically −300 to −200 kJ/mol) originating from largely unstable initial states having a H atom (radical) in the gas phase. Thus, the E–R-type process with an atomic hydrogen is significantly preferable kinetically and thermodynamically to the conventional L–H-type process. The barriers for simple H addition (1, 4, 5, 7, 8) were lower than those for dehydroxylation (3, 6), which may be because the former requires little motion of the adsorbate molecule during the hydrogen approach (see Figure S13 for details). The highest Δ*H*^‡^ of 48.4 kJ/mol was seen in the br-CH_2_OH* dehydroxylation to h-CH_2_* (6), indicating that this step is rate-determining. The entropy term tells us more about the reaction mechanism for H addition. The dehydroxylation steps (3, 6) showed significantly negative activation entropies (Δ*S*^‡^: −156.2 and −153.9 J/mol·K), which can be attributed to the loss of translational entropy of H presenting in the gas phase at the IS. However, their reaction entropies were positive (Δ*S*: 39.6 and 56.9 J/mol·K), which is explained by gaining rotational and translational entropies of H_2_O released to the gas phase. Conversely, the simple H addition steps (4, 5, 7) showed minor Δ*S*^‡^ close to zero, whereas their Δ*S* values were largely negative. This is because the TSs appear very early with low barriers (i.e., near barrierless), so that translational entropy is completely lost in each FS, whereas it remains at each TS. Importantly, the calculated Δ*H*^‡^ and Δ*S*^‡^ values in the rate-determining (48.4 kJ/mol and −153.9 J/mol·K) step are consistent with the experimental vales estimated from the Eyring plot (54.7 kJ/mol and −164.4 J/mol·K: Figure 2f), demonstrating the validity of our DFT calculation and the reaction mechanism based on E–R-type fashion. Thus, the DFT calculation corroborated the experimental findings that PDAH plays a critical role in promoting CO_2_ methanation by activating E–R-type reactions.

## Conclusions

In summary, we observed that the CO_2_ methanation over Ni/Al_2_O_3_ can be efficiently enhanced by plasma compared to thermal conditions at low temperatures below 300 °C. To understand the contribution of the plasma, it was investigated in detail, combining kinetic studies, *in situ* TIR, *in situ* XAFS, and DFT calculations. b-HCOO* is a key intermediate for CH_4_ formation, and b-HCOO* hydrogenation under thermal conditions has a high activation barrier, resulting in unsatisfactory activity. Interestingly, PDAH activates the E–R-type reaction channel, significantly lowering the energy barrier for b-HCOO* hydrogenation and leading to enhanced low-temperature CO_2_ methanation. Overall, this study highlights the role of PDAH in catalytic CO_2_ hydrogenation and demonstrates its importance. Furthermore, this finding is likely to be generalizable to other types of catalytic hydrogenation including methanol, hydrocarbon, and ammonia synthesis.

## Supporting Information Available

Additional experimental details and methods, including photographs of the experimental setup; XRD pattern and HAADF-STEM image of Ni/Al_2_O_3_; *in situ* TIR spectra of Ni/Al_2_O_3_; *in situ* Ni K-edge XAFS of Ni/Al_2_O_3_; and configurations of DFT calculations (PDF)

## References

[ref1] Saeidi S.; Najari S.; Hessel V.; Wilson K.; Keil F. J.; Concepción P.; Suib S. L.; Rodrigues A. E. Recent advances in CO2 hydrogenation to value-added products—Current challenges and future directions. Prog. Energy Combust. Sci. 2021, 85, 100905 10.1016/j.pecs.2021.100905.

[ref2] Tébar-Soler C.; Martin-Diaconescu V.; Simonelli L.; Missyul A.; Perez-Dieste V.; Villar-García I. J.; Brubach J. B.; Roy P.; Haro M. L.; Calvino J. J.; Concepción P.; Corma A. Low-oxidation-state Ru sites stabilized in carbon-doped RuO2 with low-temperature CO2 activation to yield methane. Nat. Mater. 2023, 22 (6), 762–768. 10.1038/s41563-023-01540-1. 37142737

[ref3] Zhu X.; Zong H.; Pérez C. J. V.; Miao H.; Sun W.; Yuan Z.; Wang S.; Zeng G.; Xu H.; Jiang Z.; Ozin G. A. Supercharged CO2 photothermal catalytic methanation: high conversion, rate, and selectivity. Angew. Chem., Int. Ed. 2023, 135 (22), e202218694 10.1002/ange.202218694. 36972170

[ref4] Ghaib K.; Ben-Fares F.-Z. Power-to-Methane: A state-of-the-art review. Renew. Sustainable Energy Rev. 2018, 81, 433–446. 10.1016/j.rser.2017.08.004.

[ref5] Xiong M.; Gao Z.; Qin Y. Spillover in heterogeneous catalysis: new insights and opportunities. ACS Catal. 2021, 11 (5), 3159–3172. 10.1021/acscatal.0c05567.

[ref6] Adamovich I.; Agarwal S.; Ahedo E.; Alves L. L.; Baalrud S.; Babaeva N.; Bogaerts A.; Bourdon A.; Bruggeman P. J.; Canal C.; Choi E. H.; Coulombe S.; Donkó Z.; Graves D. B.; Hamaguchi S.; Hegemann D.; Hori M.; Kim H. H.; Kroesen G. M. W.; Kushner M. J.; Laricchiuta A.; Li X.; Magin T. E.; Mededovic Thagard S.; Miller V.; Murphy A. B.; Oehrlein G. S.; Puac N.; Sankaran R. M.; Samukawa S.; Shiratani M.; Šimek M.; Tarasenko N.; Terashima K.; Thomas E. Jr.; Trieschmann J.; Tsikata S.; Turner M. M.; van der Walt I. J.; van de Sanden M. C. M.; von Woedtke T. The 2022 Plasma Roadmap: low temperature plasma science and technology. J. Phys. D: Appl. Phys. 2022, 55 (37), 373001 10.1088/1361-6463/ac5e1c.

[ref7] George A.; Shen B.; Craven M.; Wang Y.; Kang D.; Wu C.; Tu X. A Review of Non-Thermal Plasma Technology: A novel solution for CO2 conversion and utilization. Renew. Sustainable Energy Rev. 2021, 135, 109702 10.1016/j.rser.2020.109702.

[ref8] Kim D.-Y.; Saito A.; Sasaki K.; Nozaki T. In situ infrared absorption probing of plasma catalysis: vibrationally-excited species induced Mars–van Krevelen type mechanism. Plasma Sources Sci. Technol. 2022, 31 (12), 124005 10.1088/1361-6595/acab28.

[ref9] Liu S.; Winter L. R.; Chen J. G. Review of plasma-assisted catalysis for selective generation of oxygenates from CO2 and CH4. ACS Catal. 2020, 10 (4), 2855–2871. 10.1021/acscatal.9b04811.

[ref10] Bogaerts A.; Tu X.; Whitehead J. C.; Centi G.; Lefferts L.; Guaitella O.; Azzolina-Jury F.; Kim H. H.; Murphy A. B.; Schneider W. F.; Nozaki T.; Hicks J. C.; Rousseau A.; Thevenet F.; Khacef A.; Carreon M. The 2020 plasma catalysis roadmap. J. Phys. D: Appl. Phys. 2020, 53 (44), 443001 10.1088/1361-6463/ab9048.

[ref11] Kim H.-H.; Abdelaziz A. A.; Teramoto Y.; Nozaki T.; Hensel K.; Mok Y.-S.; Saud S.; Nguyen D. B.; Lee D. H.; Kang W. S. Interim report of plasma catalysis: footprints in the past and blueprints for the future. Int. J. Plasma Environ. Sci. Technol. 2021, 15 (1), e01004 10.34343/ijpest.2021.15.e01004.

[ref12] Nozaki T.; Kim D.-Y.; Chen X. Plasma-enabled electrification of chemical processes toward decarbonization society. Jpn. J. Appl. Phys. 2024, 63, 030101 10.35848/1347-4065/ad280f.

[ref13] Xu S.; Chansai S.; Xu S.; Stere C. E.; Jiao Y.; Yang S.; Hardacre C.; Fan X. CO poisoning of Ru catalysts in CO2 hydrogenation under thermal and plasma conditions: a combined kinetic and diffuse reflectance infrared fourier transform spectroscopy–mass spectrometry study. ACS Catal. 2020, 10 (21), 12828–12840. 10.1021/acscatal.0c03620.

[ref14] Sun Y.; Wu J.; Wang Y.; Li J.; Wang N.; Harding J.; Mo S.; Chen L.; Chen P.; Fu M.; Ye D.; Huang J.; Tu X. Plasma-catalytic CO2 hydrogenation over a Pd/ZnO catalyst: In situ probing of gas-phase and surface reactions. JACS Au 2022, 2 (8), 1800–1810. 10.1021/jacsau.2c00028. 36032530 PMC9400056

[ref15] Wang Y.; Yang W.; Xu S.; Zhao S.; Chen G.; Weidenkaff A.; Hardacre C.; Fan X.; Huang J.; Tu X. Shielding protection by mesoporous catalysts for improving plasma-catalytic ambient ammonia synthesis. J. Am. Chem. Soc. 2022, 144 (27), 12020–12031. 10.1021/jacs.2c01950. 35731953 PMC9284550

[ref16] Rouwenhorst K. H. R.; Engelmann Y.; van ‘t Veer K.; Postma R. S.; Bogaerts A.; Lefferts L. Plasma-driven catalysis: green ammonia synthesis with intermittent electricity. Green Chem. 2020, 22 (19), 6258–6287. 10.1039/D0GC02058C.

[ref17] Ahmad F.; Lovell E. C.; Masood H.; Cullen P. J.; Ostrikov K. K.; Scott J. A.; Amal R. Low-temperature CO2 methanation: synergistic effects in plasma-Ni hybrid catalytic system. ACS Sustain. Chem. Eng. 2020, 8 (4), 1888–1898. 10.1021/acssuschemeng.9b06180.

[ref18] Chen H.; Mu Y.; Shao Y.; Chansai S.; Xu S.; Stere C. E.; Xiang H.; Zhang R.; Jiao Y.; Hardacre C.; Fan X. Coupling non-thermal plasma with Ni catalysts supported on BETA zeolite for catalytic CO 2 methanation. Catal. Sci. Technol. 2019, 9 (15), 4135–4145. 10.1039/C9CY00590K.

[ref19] Lefferts L. Leveraging expertise in thermal catalysis to understand plasma catalysis. Angew. Chem., Int. Ed. 2024, 136 (10), e202305322 10.1002/ange.202305322. 38279548

[ref20] Prins R. Eley–Rideal, the other mechanism. Top. Catal. 2018, 61 (9), 714–721. 10.1007/s11244-018-0948-8.

[ref21] Du Y.; Tamura K.; Moore S.; Peng Z.; Nozaki T.; Bruggeman P. J. CO (B 1 Σ+→ A 1 Π) angstrom system for gas temperature measurements in CO 2 containing plasmas. Plasma Chem. Plasma Process. 2017, 37, 29–41. 10.1007/s11090-016-9759-5.

[ref22] Taguchi T.; Ozawa T.; Yashiro H. REX2000: yet another XAFS analysis package. Phys. Scr. 2005, 2005 (T115), 205 10.1238/Physica.Topical.115a00205.

[ref23] Ankudinov A. L.; Ravel B.; Rehr J.; Conradson S. Real-space multiple-scattering calculation and interpretation of x-ray-absorption near-edge structure. Phys. Rev. B 1998, 58 (12), 7565 10.1103/PhysRevB.58.7565.

[ref24] Hamilton W. C. Significance tests on the crystallographic R factor. Acta Crystallogr. 1965, 18 (3), 502–510. 10.1107/S0365110X65001081.

[ref25] Segall M.; Lindan P. J.; Probert M. a.; Pickard C. J.; Hasnip P. J.; Clark S.; Payne M. First-principles simulation: ideas, illustrations and the CASTEP code. J. Phys.: Condens. Matter 2002, 14 (11), 2717 10.1088/0953-8984/14/11/301.

[ref26] Tkatchenko A.; Scheffler M. Accurate molecular van der Waals interactions from ground-state electron density and free-atom reference data. Physical review letters 2009, 102 (7), 073005 10.1103/PhysRevLett.102.073005. 19257665

[ref27] Hu K.; Wu M.; Hinokuma S.; Ohto T.; Wakisaka M.; Fujita J.-i.; Ito Y. Boosting electrochemical water splitting via ternary NiMoCo hybrid nanowire arrays. J. Mater. Chem. A 2019, 7 (5), 2156–2164. 10.1039/C8TA11250A.

[ref28] Govind N.; Petersen M.; Fitzgerald G.; King-Smith D.; Andzelm J. A generalized synchronous transit method for transition state location. Comput. Mater. Sci. 2003, 28 (2), 250–258. 10.1016/S0927-0256(03)00111-3.

[ref29] Halgren T. A.; Lipscomb W. N. The synchronous-transit method for determining reaction pathways and locating molecular transition states. Chem. Phys. Lett. 1977, 49 (2), 225–232. 10.1016/0009-2614(77)80574-5.

[ref30] Nozaki T.; Okazaki K. Non-thermal plasma catalysis of methane: Principles, energy efficiency, and applications. Catal. Today 2013, 211, 29–38. 10.1016/j.cattod.2013.04.002.

[ref31] Koschany F.; Schlereth D.; Hinrichsen O. On the kinetics of the methanation of carbon dioxide on coprecipitated NiAl (O) x. Appl. Catal., B 2016, 181, 504–516. 10.1016/j.apcatb.2015.07.026.

[ref32] Le T. A.; Kim J.; Kang J. K.; Park E. D. CO and CO2 methanation over Ni/Al@ Al2O3 core–shell catalyst. Catal. Today 2020, 356, 622–630. 10.1016/j.cattod.2019.09.028.

[ref33] Garbarino G.; Bellotti D.; Riani P.; Magistri L.; Busca G. Methanation of carbon dioxide on Ru/Al2O3 and Ni/Al2O3 catalysts at atmospheric pressure: catalysts activation, behaviour and stability. Int. J. Hydrogen Energy 2015, 40 (30), 9171–9182. 10.1016/j.ijhydene.2015.05.059.

[ref34] Xin H.; Lin L.; Li R.; Li D.; Song T.; Mu R.; Fu Q.; Bao X. Overturning CO2 hydrogenation selectivity with high activity via reaction-induced strong metal–support interactions. J. Am. Chem. Soc. 2022, 144 (11), 4874–4882. 10.1021/jacs.1c12603. 35258951

[ref35] Prabhakar J. K.; Apte P. A.; Deo G. The kinetics of Ni/Al2O3 and Ni-Fe/Al2O3 catalysts for the CO2 methanation reaction and the reasons for promotion. Chem. Eng. J. 2023, 471, 144252 10.1016/j.cej.2023.144252.

[ref36] Kim D. Y.; Ham H.; Chen X.; Liu S.; Xu H.; Lu B.; Furukawa S.; Kim H. H.; Takakusagi S.; Sasaki K.; Nozaki T. Cooperative catalysis of vibrationally excited CO2 and alloy catalyst breaks the thermodynamic equilibrium limitation. J. Am. Chem. Soc. 2022, 144 (31), 14140–14149. 10.1021/jacs.2c03764. 35862699

[ref37] Sheng Z.; Kim H.-H.; Yao S.; Nozaki T. Plasma-chemical promotion of catalysis for CH 4 dry reforming: unveiling plasma-enabled reaction mechanisms. Phys. Chem. Chem. Phys. 2020, 22 (34), 19349–19358. 10.1039/D0CP03127E. 32822443

[ref38] Wang Z.; Zhang Y.; Neyts E. C.; Cao X.; Zhang X.; Jang B. W.-L.; Liu C.-j. Catalyst preparation with plasmas: how does it work?. ACS Catal. 2018, 8 (3), 2093–2110. 10.1021/acscatal.7b03723.

[ref39] De Bie C.; van Dijk J.; Bogaerts A. CO2 hydrogenation in a dielectric barrier discharge plasma revealed. J. Phys. Chem. C 2016, 120 (44), 25210–25224. 10.1021/acs.jpcc.6b07639.

[ref40] Lin W.; Stocker K. M.; Schatz G. C. Mechanisms of hydrogen-assisted CO2 reduction on nickel. J. Am. Chem. Soc. 2017, 139 (13), 4663–4666. 10.1021/jacs.7b01538. 28323422

[ref41] Duyar M. S.; Ramachandran A.; Wang C.; Farrauto R. J. Kinetics of CO2 methanation over Ru/γ-Al2O3 and implications for renewable energy storage applications. J. CO2 Util. 2015, 12, 27–33. 10.1016/j.jcou.2015.10.003.

[ref42] Wang X.; Shi H.; Kwak J. H.; Szanyi J. Mechanism of CO2 hydrogenation on Pd/Al2O3 catalysts: kinetics and transient DRIFTS-MS studies. ACS Catal. 2015, 5 (11), 6337–6349. 10.1021/acscatal.5b01464.

[ref43] Ano T.; Tsubaki S.; Liu A.; Matsuhisa M.; Fujii S.; Motokura K.; Chun W.-J.; Wada Y. Probing the temperature of supported platinum nanoparticles under microwave irradiation by in situ and operando XAFS. Commun. Chem. 2020, 3 (1), 86 10.1038/s42004-020-0333-y. 36703448 PMC9814256

