# Supplementary material for: Plasma-Derived Atomic Hydrogen Enables Eley–Rideal-Type CO2 Methanation at Low Temperatures
Source: JACS Au. 2024 Nov 19;5(1):169–77. doi: 10.1021/jacsau.4c00857 (PMC11775702; doi:10.1021/jacsau.4c00857)
Supplement: Supplementary file 1 — au4c00857_si_001.pdf [file au4c00857_si_001.pdf]

Supporting Information

Plasma-derived atomic hydrogen enables Eley−Rideal-type CO_2 methanation at low temperature

Dae-Yeong Kim,*,† Yoshinobu Inagaki,‡ Tsukasa Yamakawa,‡ Bang Lu,§ Yoshiaki Sato,§ Naoki Shirai,‡ Shinya Furukawa,*,# Hyun-Ha Kim,¶ Satoru Takakusagi,§ Koichi Sasaki,‡ and Tomohiro Nozaki*,†

†Department of Mechanical Engineering, Tokyo Institute of Technology, Tokyo 152-8550, Japan

‡Division of Applied Quantum Science and Engineering, Hokkaido University, Sapporo 060-8628, Japan

§Institute for Catalysis, Hokkaido University, Sapporo 001-0021, Japan

#Division of Applied Chemistry, Osaka University, Osaka 565-0871, Japan

¶National Institute of Advanced Industrial Science and Technology, Tsukuba 305-8569, Japan

*Corresponding authors. Email: nozaki.t.ab@m.titech.ac.jp (T.N.), furukawa@chem.eng.osaka-u.ac.jp (S.F.), kim.d.as@m.titech.ac.jp (D-Y.K.)

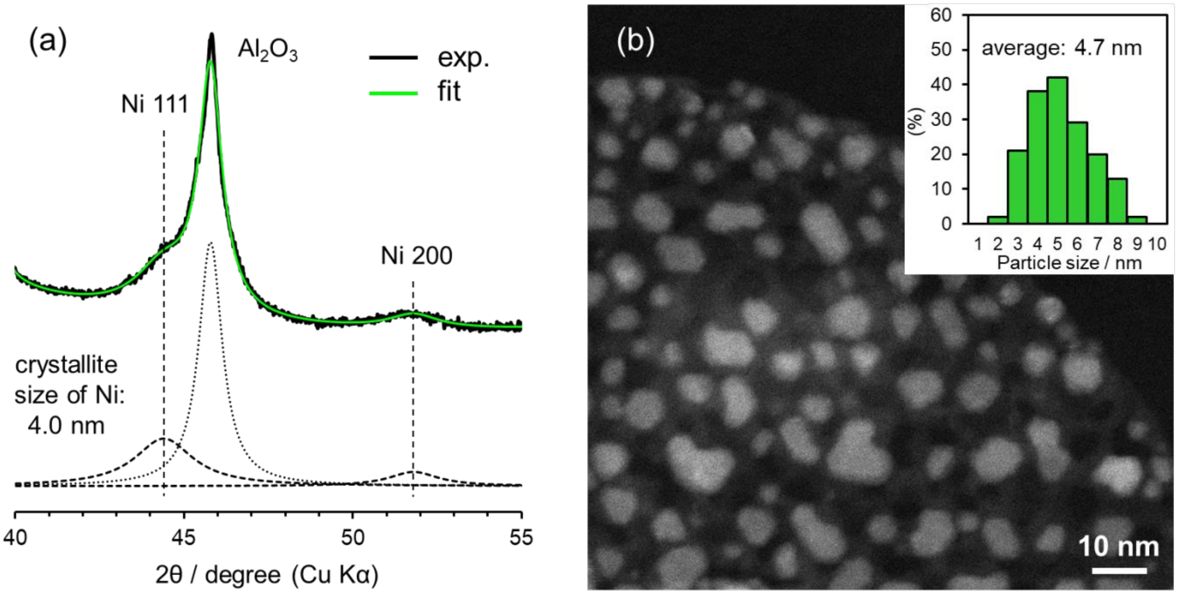

Figure S1. (a) XRD pattern and (b) HAADF-STEM image of Ni/Al_2O_3. Inset in (b) shows the size distribution of Ni nanoparticles. The average size is consistent with the crystallite size estimated by Scherrer's equation.

Calculating flow rate from QMS

The experimental setup for calculating the flow rate from QMS was configured as shown in Figure S2. Ar was set as a bypass gas to act as the QMS internal standard gas, and the mol flow rate of each gas was calculated using the following equation:

$$\frac{I_j}{I_{Ar}} = \frac{\alpha_j}{\alpha_{Ar}} \times \frac{F_j}{F_{Ar}}$$

Here j, I, α, F respectively represent a type of gas (CO_2, H_2, CH_4, CO, Ar), ion current obtained from the QMS, sensitivity, and mol flow rate. The α was calculated by flowing each gas under conditions where no reaction occurred.

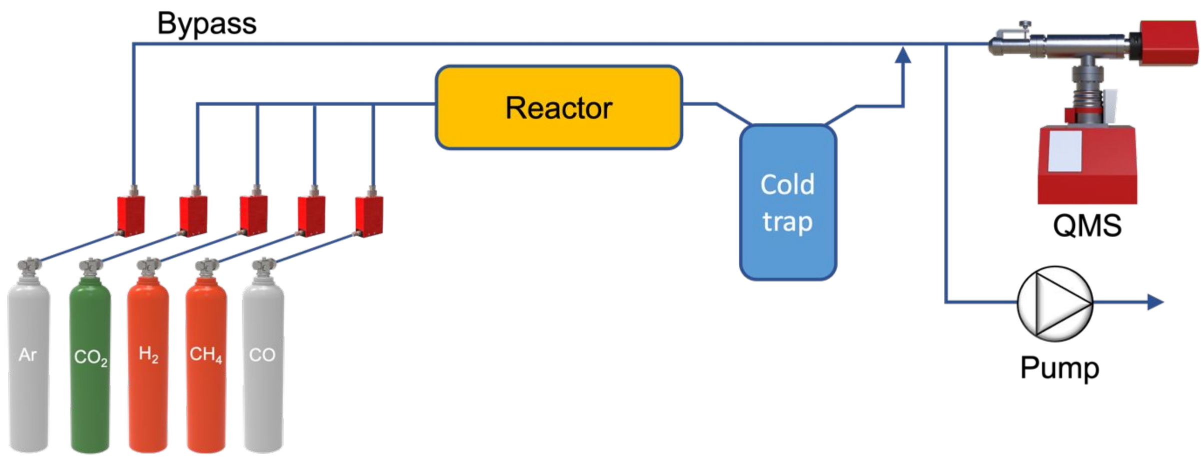

Figure S2. Schematic diagram of the experimental setup for calculating flow rate from QMS.

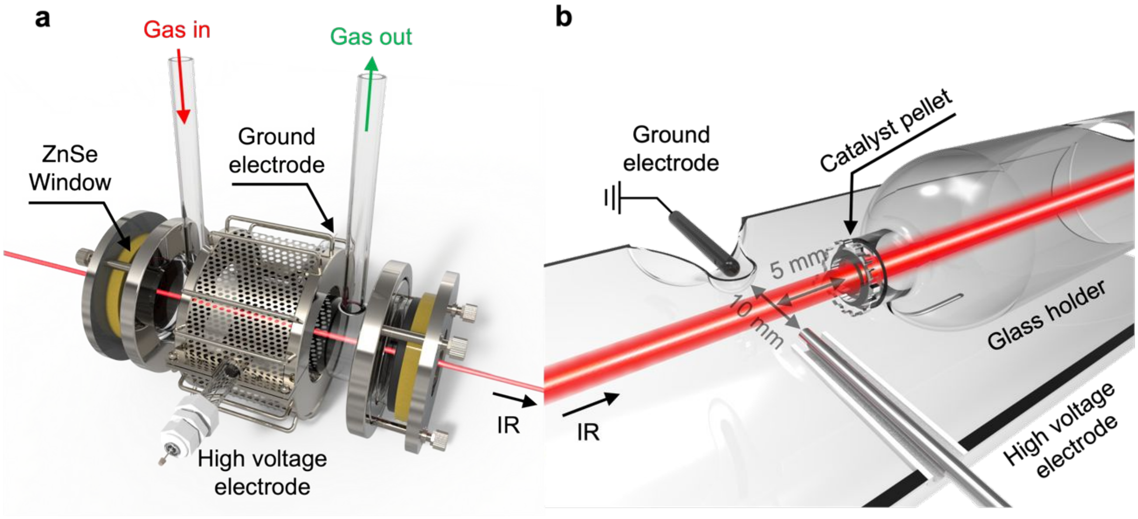

Figure S3. Schematic diagram of the *in situ* TIR cell (a) and the electrode and catalyst pellet geometry (b). The ZnSe windows were replaced with Kapton film (polymide) when *in situ* XAFS was employed

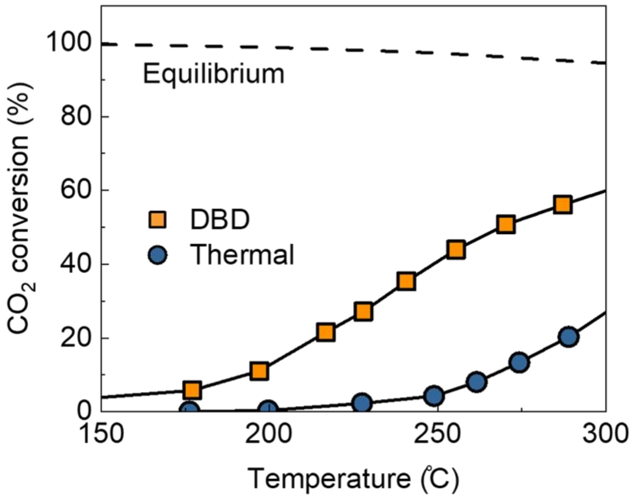

Figure S4. Temperature dependent CO_2 conversion. The dotted line indicates the equilibrium CO_2 conversion for the CO_2 methanation (H_2/CO_2 molar ratio of 4) at 80 kPa.

Kinetic analysis

CH_4 reaction rate was expressed by the power-law kinetics (Eq. (1)). Take the natural logarithm of Eq. (1) and rewrite it into Eqs. (2) - (4):

$$-\frac{dP_{CH_4}}{dt} = r_{CH_4} = k_f P_{CO_2}{}^{\alpha} P_{H_2}{}^{\beta} \quad (1)$$

$$r_{CH_4} = \ln k_f + \alpha \ln P_{CO_2} + \beta \ln P_{H_2} \quad (2)$$

$$\ln \frac{r_{CH_4}}{P_{H_2}{}^{\beta}} \propto \alpha \ln P_{CO_2} \quad (3)$$

$$\ln \frac{r_{CH_4}}{P_{CO_2}{}^{\alpha}} \propto \beta \ln P_{H_2} \quad (4)$$

Here, k_f and P expresses the reaction rate constant and the average concentration of CO_2 and H_2. α and β represent the modified reaction order for CO_2 and H_2, respectively. α and β were unknown figures at this point: Assume arbitrary values for α and β and performed iterative calculations until the deviation of α and β becomes smaller than 1% error. The feasibility of this method had been verified by comparing with conventional approach with inert gas dilution in our previous research on dry methane reforming.[1]

Reaction order was estimated from Figure 2d and 2e at fixed total flow rate while varying H_2/CO_2 ratio without dilution gas. Figure S2 represents the corresponding CO_2 and H_2 conversion and CH_4 selectivity. Experimental conditions are provided in the figure caption of Figure S2. In Figure 2d and 2e, $\ln\frac{r_{CH_4}}{P_{H_2}{}^{\beta}}$ v.s. $\ln P_{CO_2}$ (Figure 2d) and $\ln\frac{r_{CH_4}}{P_{CO_2}{}^{\alpha}}$ v.s. $\ln P_{H_2}$ (Figure 2e) express the linear relationship.

Apparent activation energy (E_{app}) under thermal and DBD conditions was determined according to Eqs. (1) and (5):

$$k_f = A e^{-\frac{E_{app}}{RT}} \quad (5)$$

The A, R and T represent pre-exponential factor, universal gas constant and catalyst temperature (K), respectively.

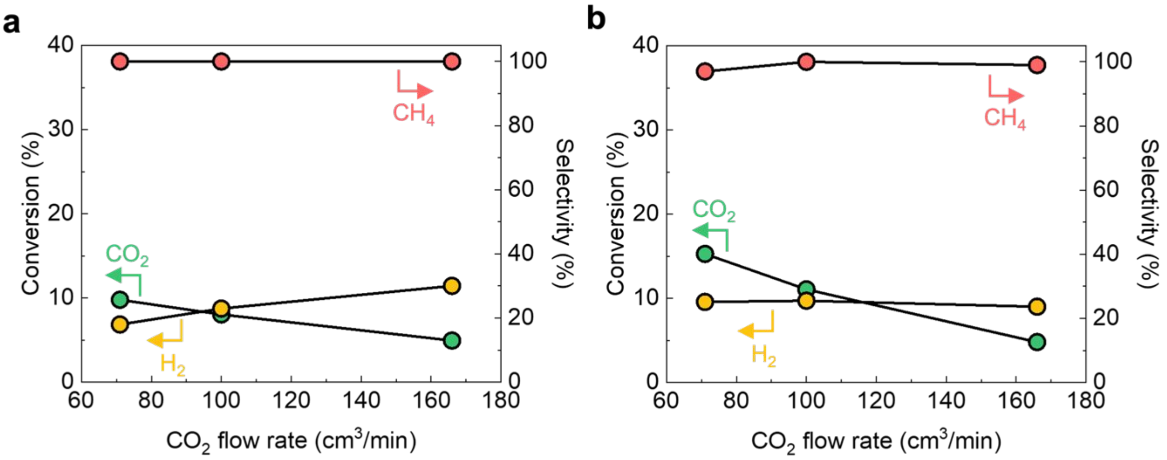

Figure S5. CO_2 and H_2 conversion (%) with respect to the initial CO2 flow rate on Ni/Al_2O_3. (a) Thermal and (b) DBD conditions. Catalyst temperature under thermal and DBD conditions were fixed at 260 and 200 °C respectively. Total flow rate = 500 mL/min (STP); H_2/CO_2 = 2, 4, and 6; WHSV = 5000 cm_3/g/h (STP); pressure = 80 kPa; and SEI = 0.6 eV/molecules. Kinetic analyses were performed in the packed-bed DBD reactor (Figure 1) without the use of Ar.

Lifetime of plasma-derived atomic hydrogen

The lifetime of plasma-derived atomic hydrogen (PDAH) in the gas phase was examined in the spatial afterglow of an atmospheric pressure DBD reported elsewhere.[2] Briefly, the plasma source was composed of a quartz tube with an inner diameter of 4 mm, a tungsten rod, and a copper ring. The tungsten rod was placed at the center of the quartz tube, and it was connected to a high-voltage pulsed power supply. The copper ring was attached on the outside of the quartz tube, and it was connected to the electrical ground (Figure S2). The voltage applied to the power supply was 14 kV (peak-to-peak). The mixture of H_2, CO_2, and Ar was fed from an end of the quartz tube. The flow rates of H_2, CO_2, and Ar were 0.1, 0.025, and 1.4 slm, respectively, corresponding to the flow velocity of 200 cm/s. The discharge with the optical emission was localized in the region with the copper ring, but the effluent from the discharge zone was ejected from the other end of the quartz tube and was transported to the spatial afterglow region. The plasma source including the spatial afterglow was installed inside a vacuum chamber.

As shown in Figure S5, the absolute density of atomic hydrogen was measured by two-photon absorption laser-induced fluorescence (TALIF). A tunable dye laser beam was focused into the spatial afterglow from the radial direction. The wavelength of the dye laser was tuned at the two-photon excitation wavelength from $1s^2S_{1/2}$ to $3d^2D_{3/2,5/2}$ states of atomic hydrogen (205.08 nm). The image of the fluorescence at the Balmer α line was captured using a charge coupled device camera with a gated image intensifier (ICCD camera). An interference filter with the transmission at the Balmer a line was placed in front of the ICCD camera to eliminate the optical emission from the discharge. The TALIF image represented the radial distribution of the atomic hydrogen density along the path of the laser beam. The axial distribution was obtained by changing the distance between the exit of the effluent and the dye laser beam. The standard calibration method using TALIF of Kr was adopted to evaluate the absolute density

of atomic hydrogen.[3] The plasma was not ignited in TALIF of Kr. Kr was admixed into the Ar flow, and the density of Kr was estimated from the flow rates of Ar and Kr. The absolute density of PDAH was deduced by comparing the fluorescence intensities of H and Kr. The cross sections of two-photon excitation, the transition probabilities of the fluorescence, the rate coefficients of collisional quenching, the transmission of the interference filter, and the quantum efficiencies of the ICCD camera at the fluorescence wavelengths were considered in the evaluation of the absolute density of PDAH.

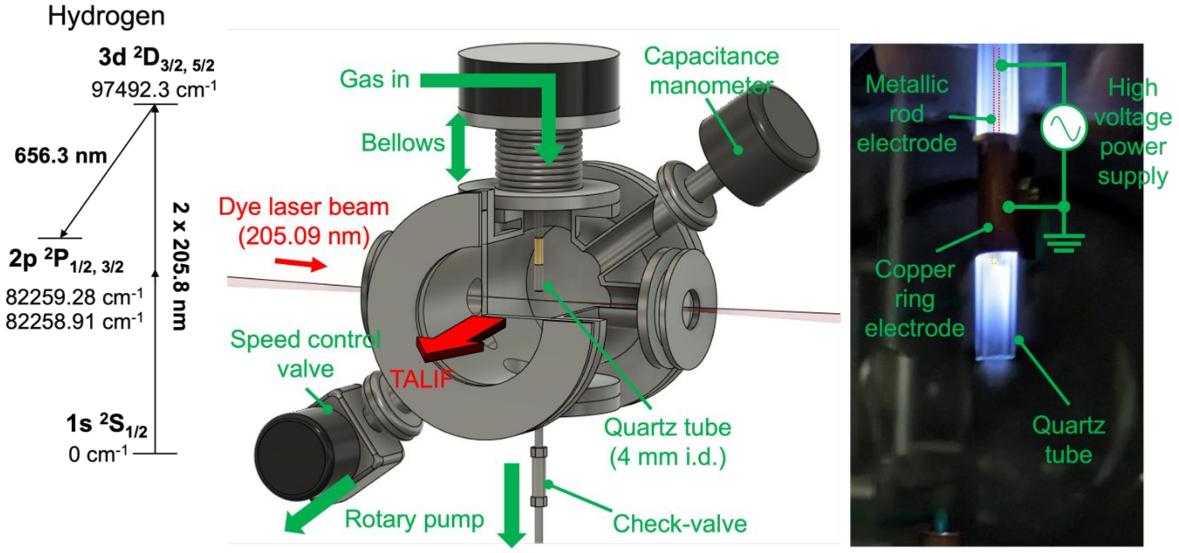

Figure S6. The detailed setup of the TALIF. The inset picture shows the DBD generated by the high-voltage power source under Ar (1.4 slm) + H_2 (0.1 slm) + CO_2 (0.025 slm).

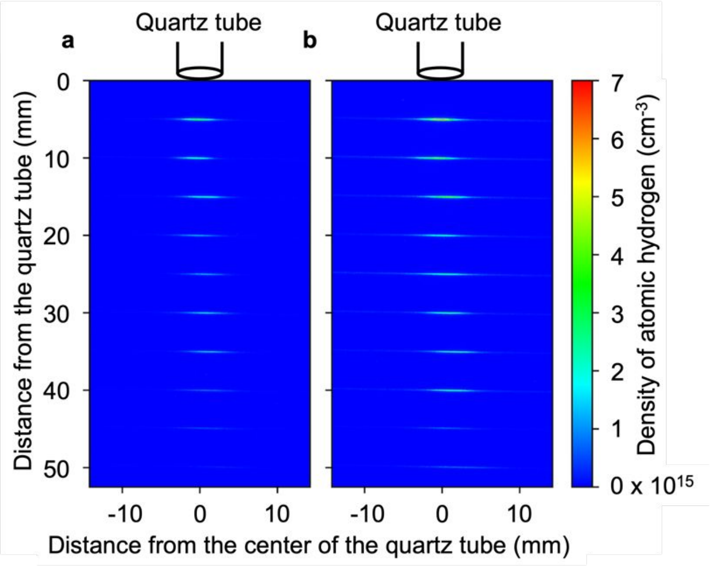

Figure S7. Laser-induced fluorescence images in the spatial afterglow of the (a) $Ar/H_2/CO_2$ and (b) Ar/H_2 discharge.

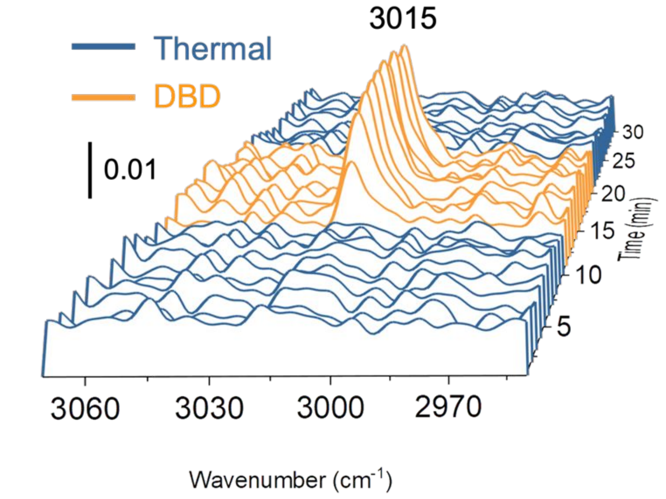

Figure S8. *In situ* TIR spectra of CH_4 peaks area (3015 cm^{-1}) under both thermal and DBD condition.

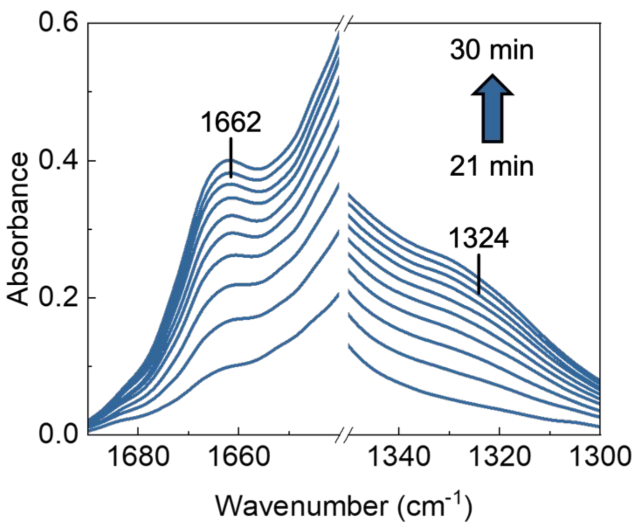

Figure S9. *In situ* TIR Spectra of the evolution of m-HCOO* (1662 and 1324 cm^{-1}) under thermal conditions. This shows the evolution of m-HCOO* when switching from DBD conditions to thermal conditions in Figure 3 of the main text.

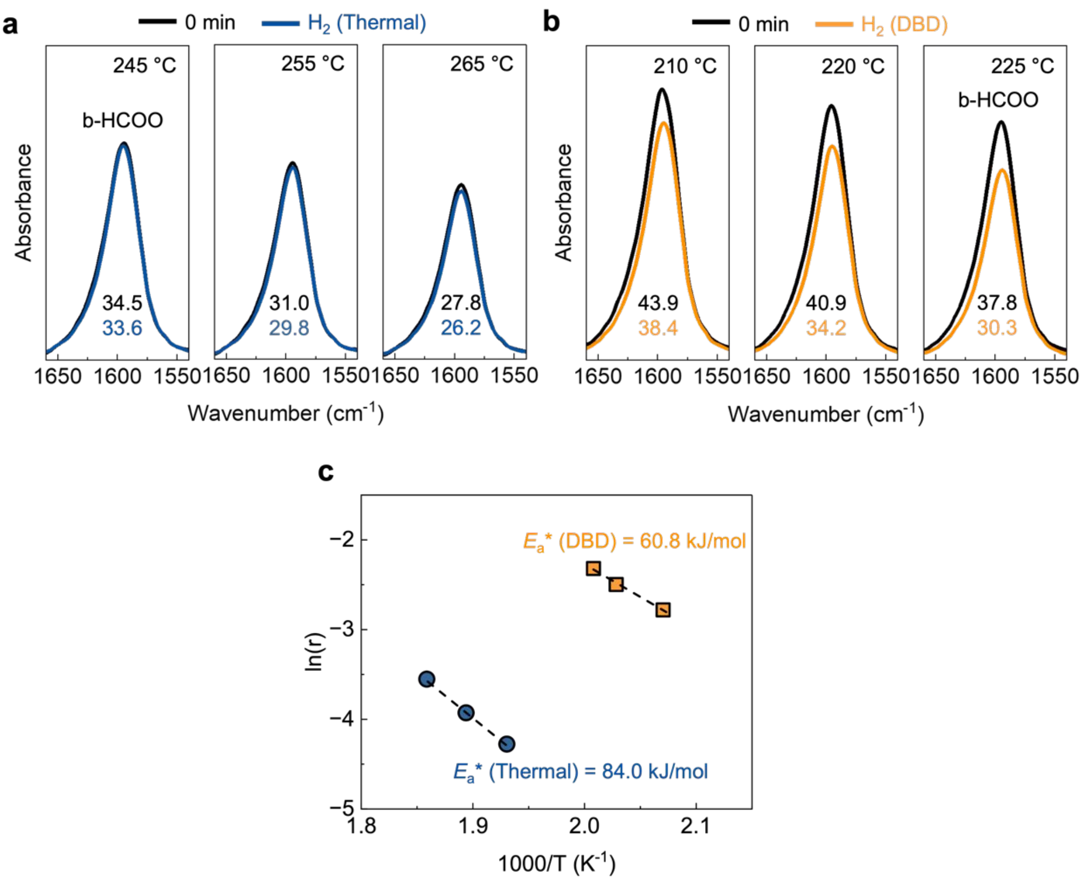

Figure S10. Reaction kinetic of b-HCOO* hydrogenation. *In situ* TIR spectra over Ni/Al_2O_3 at 210 °C after switching feed gas from (a) A CO_2+H_2 mixture (H_2/CO_2 = 4) under thermal conditions to Ar under thermal conditions to H_2 under thermal conditions and (b) A CO_2+H_2 mixture (H_2/CO_2 = 4) under thermal conditions to Ar under thermal conditions to H_2 under DBD conditions. (c) Arrhenius plots of b-HCOO* hydrogenation.

To investigate the kinetics of b-HCOO* hydrogenation, b-HCOO* was formed by flowing the CO_2+H_2 mixture for 10 min under thermal conditions at 210 °C. Then, after purging with Ar, H_2 was flowed for 2 min under thermal or DBD conditions at various temperatures. Figures S9a and b show the TIR spectra of the b-HCOO* peak area (1660-1540 cm^{-1}) before and after flowing H_2 under Thermal and DBD conditions, respectively. The numbers in each figure represent the peak area. Figure S9c shows the Arrhenius plots of the reduction rate (hydrogenation rate) of the b-HCOO* peak area after flowing H_2 in Figures S9a and b.

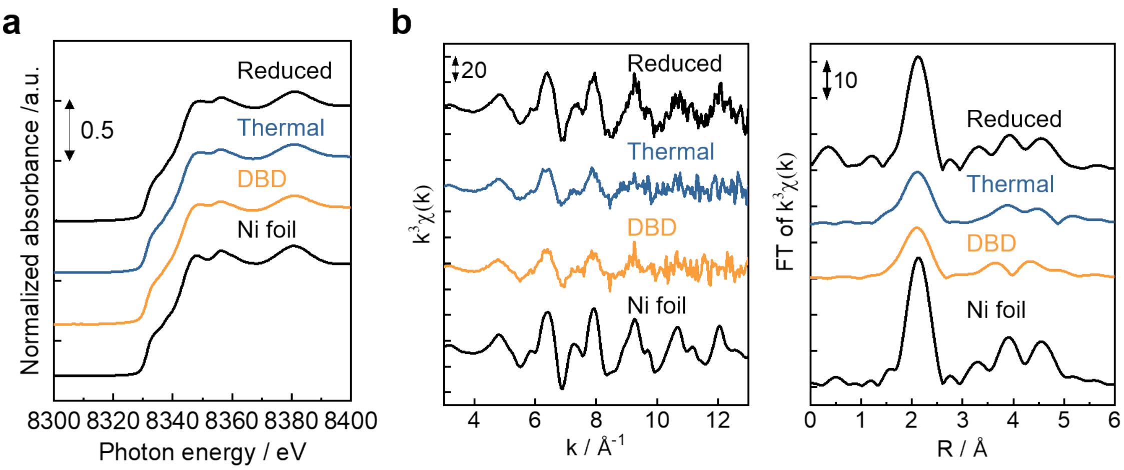

Figure S11. *In situ* Ni K-edge XAFS of the Ni/Al_2O_3. (a) XANES spectra measured after H_2 reduction at 700 °C cooling at RT during CO_2 methanation under thermal and DBD conditions at 210 °C and Ni foil measured at RT. (b) Corresponding EXAFS and Fourier-transformed (FT) EXAFS spectra.

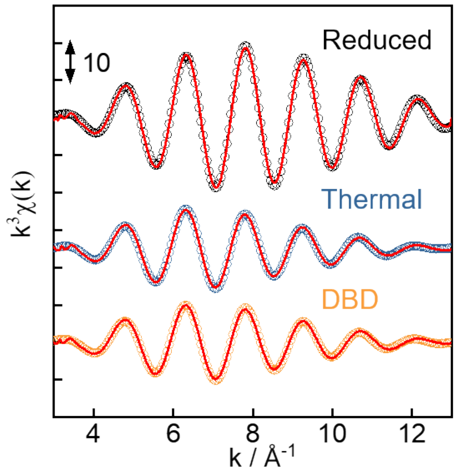

Figure S12. EXAFS fitting curves for the Ni/Al_2O_3 after H_2 reduction at 700 °C and cooling at RT during CO_2 methanation under thermal and DBD conditions at 210 °C. The calculated curves are shown as the red solid lines. See the Table S2 for the details of the fitting.

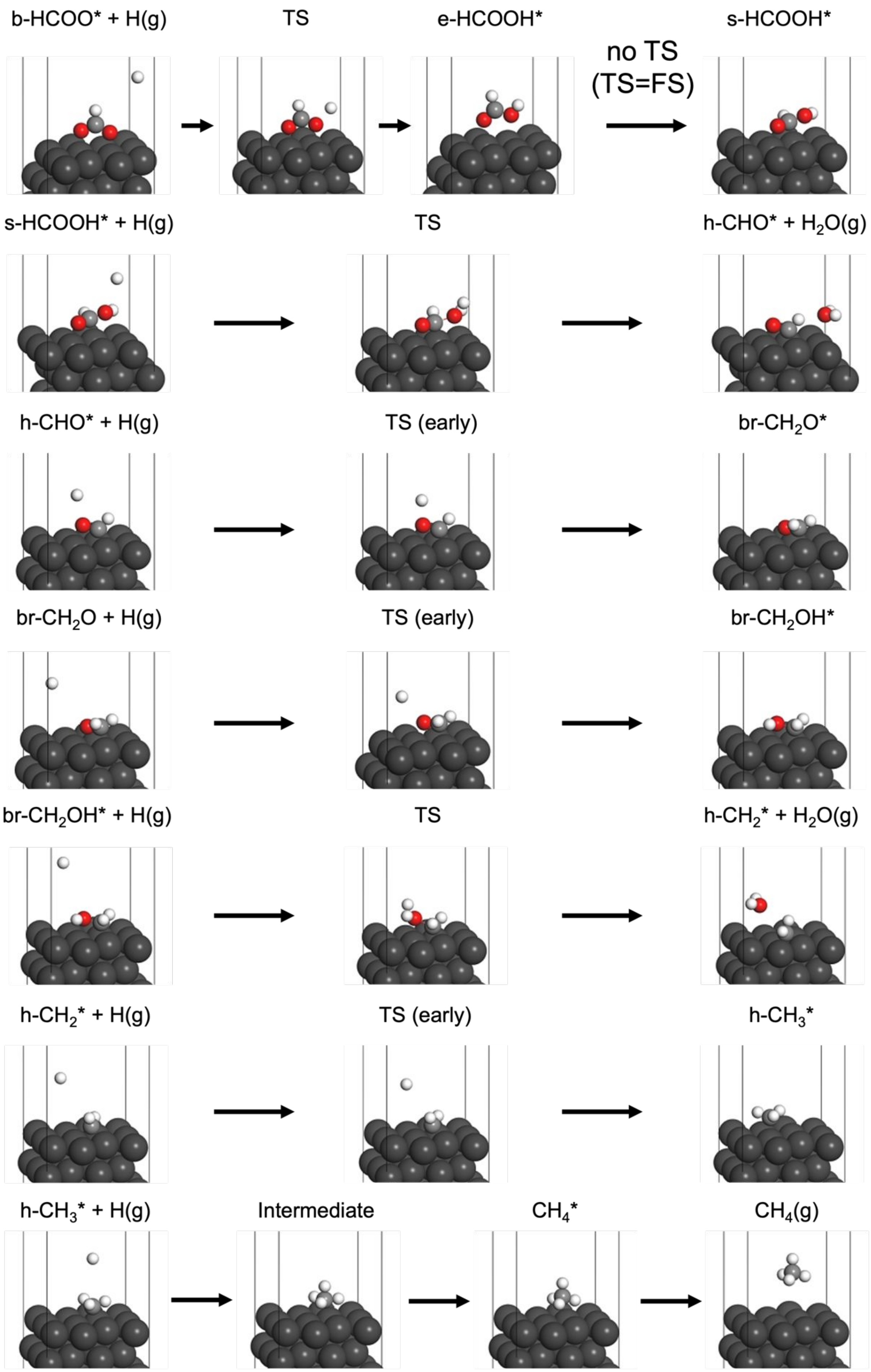
b-HCOO* + H(g)
TS
e-HCOOH*
no TS
(TS=FS)
s-HCOOH*
s-HCOOH* + H(g)
TS
h-CHO* + H_2O(g)
h-CHO* + H(g)
TS (early)
br-CH_2O*
br-CH_2O + H(g)
TS (early)
br-CH_2OH*
br-CH_2OH* + H(g)
TS
h-CH_2* + H_2O(g)
h-CH_2* + H(g)
TS (early)
h-CH_3*
h-CH_3* + H(g)
Intermediate
CH_4*
CH_4(g)

Figure S13. The configurations of initial, transition and final state for CO_2 methanation over Ni(111) in Eley−Rideal-type reactions. Color scheme: black, nickel; gray, carbon; red, oxygen; and white, hydrogen.

Table S1. Assignments of the peaks in the TIR spectra of surface species.

**Assignment**	**Vibration mode**	**Wavenumber (cm^{-1})**	**Ref**
CO^*	v(C-O) linear	2000 ~ 2060	[4]
	v(C-O) bridge	1900 ~ 2000	
	v(C-O) hollow	1830 ~ 1900	
HCO_3^*	v_a (O-C-O)	1649	[5,6]
	v_s (O-C-O)	1446	
	δ (OH)	1230	
m-$HCOO^*$	v_a (O-C-O)	1662	[7,8]
	v_s (O-C-O)	1324	
b-$HCOO^*$	v_a (O-C-O)	1595	[9,10]
	δ (CH)	1392	
	v_s (O-C-O)	1378	
CH_4	δ (CH)	3015	[11]
	v (CH)	1305	

Table S2. Curve fitting results of the Ni K-edge EXAFS spectra for the Ni/Al_2O_3.

Conditions	Shell	N_1	r_1/Å	ΔE_1/eV	$\sigma_1^2/10^{-3}$ Å^2	*R*-factor/%[a]
Reduced	Ni-Ni	11.6 ± 0.9	2.48 ± 0.01	-0.7 ± 0.9	4.4 ± 0.5	0.3
Thermal	Ni-Ni	11.4 ± 0.9	2.49 ± 0.01	-0.1 ± 1.0	10.0 ± 0.5	0.4
DBD	Ni-Ni	10.2 ± 0.9	2.48 ± 0.01	-1.4 ± 1.0	9.4 ± 0.8	0.5

[a] Goodness of the fit between the observed and calculated data was illustrated by the EXAFS fitting curves shown in Figure S12.

Table S3. Fractional coordinates of Ni atoms in the optimized bare supercell

No.	a	b	c	No.	a	b	c	No.	a	b	c
Ni(1)	0.25006	0.83334	0.17722	Ni(25)	0.25006	0.16667	0.17722	Ni(49)	0.25006	0.50001	0.17722
Ni(2)	0.24998	0.83334	0.44257	Ni(26)	0.24998	0.16667	0.44257	Ni(50)	0.24998	0.50000	0.44257
Ni(3)	0.16682	0.00002	0.09008	Ni(27)	0.16682	0.33335	0.09008	Ni(51)	0.16682	0.66669	0.09008
Ni(4)	0.16652	0.99997	0.35238	Ni(28)	0.16652	0.33331	0.35238	Ni(52)	0.16652	0.66664	0.35238
Ni(5)	0.08323	0.16668	0.00267	Ni(29)	0.08323	0.50001	0.00267	Ni(53)	0.08323	0.83335	0.00271
Ni(6)	0.08339	0.16665	0.26461	Ni(30)	0.08339	0.49998	0.26461	Ni(54)	0.08339	0.83331	0.26461
Ni(7)	0.50007	0.66668	0.17722	Ni(31)	0.50007	0.00001	0.17722	Ni(55)	0.50007	0.33334	0.17722
Ni(8)	0.49998	0.66667	0.44257	Ni(32)	0.49999	0.00000	0.44257	Ni(56)	0.49999	0.33334	0.44257
Ni(9)	0.41682	0.83335	0.09009	Ni(33)	0.41682	0.16669	0.09009	Ni(57)	0.41682	0.50002	0.09009
Ni(10)	0.41651	0.83331	0.35237	Ni(34)	0.41651	0.16664	0.35237	Ni(58)	0.41651	0.49997	0.35237
Ni(11)	0.33323	0.00002	0.00269	Ni(35)	0.33323	0.33335	0.00269	Ni(59)	0.33323	0.66668	0.00269
Ni(12)	0.33339	0.99998	0.26460	Ni(36)	0.33339	0.33331	0.26460	Ni(60)	0.33339	0.66665	0.26460
Ni(13)	0.75006	0.50001	0.17722	Ni(37)	0.75006	0.83334	0.17722	Ni(61)	0.75006	0.16668	0.17722
Ni(14)	0.74998	0.50000	0.44257	Ni(38)	0.74998	0.83334	0.44257	Ni(62)	0.74998	0.16667	0.44257
Ni(15)	0.66682	0.66669	0.09008	Ni(39)	0.66682	0.00002	0.09009	Ni(63)	0.66682	0.33335	0.09009
Ni(16)	0.66652	0.66664	0.35238	Ni(40)	0.66652	0.99997	0.35238	Ni(64)	0.66652	0.33330	0.35238
Ni(17)	0.58323	0.83335	0.00267	Ni(41)	0.58323	0.16668	0.00271	Ni(65)	0.58323	0.50001	0.00267
Ni(18)	0.58339	0.83331	0.26461	Ni(42)	0.58339	0.16665	0.26461	Ni(66)	0.58339	0.49998	0.26461
Ni(19)	0.00007	0.33334	0.17722	Ni(43)	0.00007	0.66668	0.17722	Ni(67)	0.00007	0.00001	0.17722
Ni(20)	0.99998	0.33334	0.44257	Ni(44)	0.99998	0.66667	0.44257	Ni(68)	0.99998	0.00001	0.44257
Ni(21)	0.91682	0.50002	0.09009	Ni(45)	0.91682	0.83335	0.09009	Ni(69)	0.91682	0.16669	0.09009
Ni(22)	0.91651	0.49997	0.35237	Ni(46)	0.91651	0.83330	0.35237	Ni(70)	0.91651	0.16664	0.35237
Ni(23)	0.83323	0.66668	0.00269	Ni(47)	0.83323	0.00001	0.00269	Ni(71)	0.83323	0.33335	0.00269
Ni(24)	0.83339	0.66664	0.26460	Ni(48)	0.83339	0.99998	0.26460	Ni(72)	0.83339	0.33331	0.26460

Table S4. Reaction scheme of CO_2 methanation over Ni(111) in Eley−Rideal reactions and the corresponding electronic activation energy ($\Delta E^{\ddagger}$) and reaction energy (ΔE).

**Chemical equation**	$\mathbf{\Delta E^{\ddagger}}$	$\mathbf{\Delta E}$
	**(kJ/mol)**	
b-$HCOO^*$ + H(g) → e-$HCOOH^*$	37.4	−210.1
e-$HCOOH^*$ → s-$HCOOH^*$	31.1	31.1
s-$HCOOH^*$ + H(g) → h-CHO^* + H_2O(g)	30.9	−324.4
h-CHO^* + H(g) → h-CH_2O^*	15.0	−234.2
h-CH_2O^* + H(g) → br-CH_2OH^*	10.0	−254.8
br-CH_2OH^* + H(g) → h-$CH_2{}^*$ + H_2O(g)	60.5	−286.7
h-$CH_2{}^*$ + H(g) → h-$CH_3{}^*$	2.8	−263.1
h-$CH_3{}^*$ + H(g) → CH_4(g)	0.0	−305.0

Abbreviation of adsorption configuration; g: gas-phase, b: bidentate, e: end-on, s: side-on, h: hollow, br: bridge

References

(1) Sheng, Z.; Watanabe, Y.; Kim, H.-H.; Yao, S.; Nozaki, T. Plasma-enabled mode-selective activation o f CH4 for dry reforming: first touch on the kinetic analysis. *Chem. Eng. J.* **2020**, *399*, 125751.

(2) Sasaki, K.; Nishiyama, S.; Shirai, N. Observation of 1D−1S forbidden optical emission of atomic oxy gen in atmospheric-pressure N2/O2 plasma jet. *Contrib. Plasma Phys.* **2020**, *60* (10), e202000061.

(3) Niemi, K.; Schulz-Von Der Gathen, V.; Döbele, H. Absolute calibration of atomic density measurem ents by laser-induced fluorescence spectroscopy with two-photon excitation. *J. Phys. Appl. Phys.* **2001**, *34* (15), 2330.

(4) Fujita, S.-i.; Nakamura, M.; Doi, T.; Takezawa, N. Mechanisms of methanation of carbon dioxide a nd carbon monoxide over nickel/alumina catalysts. *Appl. Catal., A* **1993**, *104* (1), 87-100.

(5) Baltrusaitis, J.; Jensen, J. H.; Grassian, V. H. FTIR spectroscopy combined with isotope labeling and quantum chemical calculations to investigate adsorbed bicarbonate formation following reaction of ca rbon dioxide with surface hydroxyl groups on Fe2O3 and Al2O3. *J. Phys. Chem. B* **2006**, *110* (24), 12 005-12016.

(6) Szanyi, J.; Kwak, J. H. Dissecting the steps of CO 2 reduction: 1. The interaction of CO and CO 2 with γ-Al 2 O 3: an in situ FTIR study. *Phys. Chem. Chem. Phys.* **2014**, *16* (29), 15117-15125.

(7) Jia, X.; Zhang, X.; Rui, N.; Hu, X.; Liu, C.-j. Structural effect of Ni/ZrO2 catalyst on CO2 methanati on with enhanced activity. *Appl. Catal., B* **2019**, *244*, 159-169.

(8) Cored, J.; Mazarío, J.; Cerdá-Moreno, C.; Lustemberg, P. G.; Ganduglia-Pirovano, M. V.; Domine, M. E.; Concepción, P. Enhanced Methanol Production over Non-promoted Cu–MgO–Al2O3 Materials with Ex-solved 2 nm Cu Particles: Insights from an Operando Spectroscopic Study. *ACS Catal.* **2022**, *12* (7), 3845-3857.

(9) Wang, J.; Li, G.; Li, Z.; Tang, C.; Feng, Z.; An, H.; Liu, H.; Liu, T.; Li, C. A highly selective and sta ble ZnO-ZrO2 solid solution catalyst for CO2 hydrogenation to methanol. *Sci. Adv.* **2017**, *3* (10), e170 1290.

(10) Kattel, S.; Yan, B.; Yang, Y.; Chen, J. G.; Liu, P. Optimizing binding energies of key intermediates for CO2 hydrogenation to methanol over oxide-supported copper. *J. Am. Chem. Soc.* **2016**, *138* (38), 12440-12450.

(11) Zhang, Z.; Shen, C.; Sun, K.; Jia, X.; Ye, J.; Liu, C.-j. Advances in studies of the structural effects of supported Ni catalysts for CO 2 hydrogenation: from nanoparticle to single atom catalyst. *J. Mate r. Chem. A* **2022**, *10* (11), 5792-5812.
